# Calmodulin-like proteins localized to the conoid regulate motility and cell invasion by *Toxoplasma gondii*

**DOI:** 10.1371/journal.ppat.1006379

**Published:** 2017-05-05

**Authors:** Shaojun Long, Kevin M. Brown, Lisa L. Drewry, Bryan Anthony, Isabelle Q. H. Phan, L. David Sibley

**Affiliations:** 1 Department of Molecular Microbiology, Washington University School of Medicine, St. Louis. Missouri, United States of America; 2 The Center for Infectious Disease Research, formerly Seattle BioMed, and Seattle Structural Genomics Center for Infectious Disease, Seattle, Washington, United States of America; University of Geneva, SWITZERLAND

## Abstract

*Toxoplasma gondii* contains an expanded number of calmodulin (CaM)-like proteins whose functions are poorly understood. Using a combination of CRISPR/Cas9-mediated gene editing and a plant-like auxin-induced degron (AID) system, we examined the roles of three apically localized CaMs. CaM1 and CaM2 were individually dispensable, but loss of both resulted in a synthetic lethal phenotype. CaM3 was refractory to deletion, suggesting it is essential. Consistent with this prediction auxin-induced degradation of CaM3 blocked growth. Phenotypic analysis revealed that all three CaMs contribute to parasite motility, invasion, and egress from host cells, and that they act downstream of microneme and rhoptry secretion. Super-resolution microscopy localized all three CaMs to the conoid where they overlap with myosin H (MyoH), a motor protein that is required for invasion. Biotinylation using BirA fusions with the CaMs labeled a number of apical proteins including MyoH and its light chain MLC7, suggesting they may interact. Consistent with this hypothesis, disruption of MyoH led to degradation of CaM3, or redistribution of CaM1 and CaM2. Collectively, our findings suggest these CaMs may interact with MyoH to control motility and cell invasion.

## Author summary

One of the most common motifs that binds calcium to transduce intracellular signals is called an EF hand- named after the globular domain structure first characterized in ovalbumin. A conserved cluster of four EF hands, each of which that binds one calcium atom, is a conserved feature of calmodulin, centrins, and calmodulin-like proteins, including myosin light chains. Although the presence of EF hands is predictive of calcium binding, it alone does not allow classification of biological function as this set of conserved proteins have very diverse functions. Here we used modified editing procedures based on CRISPR/Cas9 combined with a plant-like degradation system to define the roles of three calmodulin-like proteins in *T*. *gondii*. These proteins all localized to a specialized apical structure called the conoid where they overlap with the motor protein called MyoH. Additionally, biochemical and genetic studies suggest they coordinately regulate cell invasion. These new genomic editing tools, combined with an efficient system for protein degradation, expand the functional tool kit for an analysis of essential genes and proteins in *T*. *gondii*.

## Introduction

*Toxoplasma gondii* is a protozoan parasite that causes widespread infection in animals and zoonotic disease in humans. Equipped with excellent techniques for forward and reverse genetics, it has become a model for examining the unique adaptations of apicomplexan parasites, which include a number of parasites that cause diseases in humans and animals including *Plasmodium*, *Cryptosporidium*, and *Eimeria*. The phylum is named for its apical complex of several specialized secretory organelles and uniquely organized cytoskeletal proteins, features that are critical for motility and cell invasion [1]. Although a number of the key proteins regulating protein secretion, motility, and cell invasion have been identified; the steps regulating these processes remain largely uncharacterized.

Gliding motility by *T*. *gondii* is a substrate-dependent process that depends on an unconventional actin that assembles into short, unstable filaments beneath the plasma membrane [2, 3]. Motility also relies on apically secreted adhesins that are discharged from micronemes and occupy the cell surface as transmembrane proteins [4]. Rearward translocation of surface adhesins by myosins, together with a complex known as the glideosome that anchors the motor to the inner membrane complex [5], is thought to be responsible for the forward motion of the parasite along substrates. TgMyoA is distributed beneath the plasma membrane over the major surface of the parasite and is the main motor for gliding motility [6]. In contrast, MyoH functions specifically at the conoid, where it is involved in translocation of adhesins from their initial apical location to the collar region, prior to their being handed off to MyoA to continue their rearward journey [7]. During translocation, a plekstrin homology and armadillo repeat domain-containing protein called the glideosome-associated connector (GAC) has been implicated in tethering the cytoplasmic tails of micronemal adhesins to the actin-cytoskeleton [8]. Actin-myosin based gliding motility is also important for cell invasion as shown by the greatly decreased efficiency in invasion when these components are depleted or blocked pharmacologically [9, 10], and the slower rate of entry among those parasites that continue to invade after inducible deletion of *ACT1* [11] or *MYOA* [12], which implies a role in force generation during entry. Following apical attachment to the host cell, the parasite also discharges the contents of rhoptries into the host cell and the vacuole that the parasite will ultimately occupy [13]. Proteins discharged from the rhoptry neck (so called RONs) form a receptor in the plasma membrane that interacts with micronemal proteins such as AMA1 to establish a junction that the parasite invades though [14]. Depletion of MyoH results in parasites that apically attach to the host cell and form a tight junction, but are unable to move into the host cell [7].

*Toxoplasma gondii* contains a specialized apical structure called the conoid [15], which is comprised of short spiral, nontubular polymers of tubulin, two preconoidal rings, and a single polar ring [16]. The conoid structure is conserved in coccidians, although it is simplified to a single apical polar ring in more distantly related members of the phylum including *Plasmodium* [15]. The conoid serves as a microtubule-organizing center to collect a series of singlet microtubules that lie beneath the cell cortex [15]. Micronemes are secreted through the center of the conoid, fusing with the plasma membrane at the apex and discharging their contents onto the surface membrane [17]. Both the extension of the conoid [18], and release of micronemes [19], are governed by increases in cytoplasmic calcium, which is released from intracellular stores [20]. Microneme secretion is regulated by calcium dependent protein kinases (CDPKs) [21, 22], which control a number of calcium dependent processes in apicomplexans [23]. CDPKs are unique in containing a kinase domain fused to a regulatory domain containing a calmodulin-like domain. The calmodulin-like domain typically contains four copies of a structural motif called an EF hand that consists of a helix (E)-loop-helix (F) topology of ~12 amino acids that are acidic, including a cluster of conserved Asp residues that bind to calcium ions [24]. In the case of canonical CDPKs, calcium binding by the four conserved EF hands dramatically alters the conformation of the calmodulin-like domain to activate the kinase [25]. Although calcium activating CDPKs provide one mechanism by which the parasite can respond to calcium, *T*. *gondii* also contains many other proteins that contain EF hand domains but which lack kinase domains.

In conventional CaM, calcium binding by the EF hands alters interactions and modulates function of many different partner proteins including motor proteins, ion channels, and other enzymes [26]. By searching for conserved orthologs of CaM-like proteins from fungi, animals and plants, an initial study identified 13 CaM-like proteins in *T*. *gondii* including a single highly conserved calmodulin (CaM), several centrins, and numerous CaM-like genes [27]. The highly conserved, conventional calmodulin (CaM) found in *T*. *gondii* is localized to the apical and basal ring and acts as a regulator of calcineurin [28]. Centrin 1 has been found to be at the centriole, and two other conserved centrins have been localized to the spindle pole and centrosome, where they presumably function in cell division, although CEN2 also localizes to the preconoidal ring and basal complex [29]. Two CaM-like proteins called CaM1 and CaM2 were previously localized to the conoid [16], however, their functions have not been examined. A separate phylogenetic study that examined the repertoire of myosin light chains (MLC), which also consist of CaM-like domains with EF hands, described 6 additional CaM-like proteins and localized them to different compartments in the parasite [30]. This phylogenetic comparison also included a 7^th^ CaM-like protein (i.e. TGME49_026040, referred to as CaM3 here), but did not examine its location or function [30]. Here we chose to examine three of these CaM-like proteins (i.e. CaM1, CaM2, and CaM3) that are enriched in the conoid proteome [16], suggesting they may control calcium-dependent processes in this unique apical structure, although they have not been functionally examined before.

Previous studies have shown that CRISPR/Cas9 provide an efficient means of gene disruption and tagging in *T*. *gondii* [31, 32]. This approach was recently extended to perform a genome wide screen that revealed the fitness defects of many genes [33], and yet systems to study essential genes have been limited. Previous studies have approached the study of essential genes using Tet-off regulated expression [6] or dimerizable Cre−mediated recombination (DiCre) [34]. However, these systems respond relatively slowly, resulting in pleomorphic phenotypes that can be hard to decipher. Previous approaches to regulate protein expression via a ligand-controlled destabilization domain [35], are also hampered by the toxicity of Shield-1, as well as relatively slow protein turnover. An alternative system involves the plant auxin induced degradation (AID) system that utilizes the highly conserved proteins Skp1 and Cullin1, together with a F-box protein ubiquitin ligase called TIR1, which is found only in plants. The plant hormone indoleacetic acid or auxin serves as an inducer promoting physical interaction of the auxin receptor-TIR1 and proteins tagged with an auxin-inducible degron (AID) domain [36]. This system has been successfully adapted to various systems including mammalian cells [37], yeast [36], and *Plasmodium* [38] [39], indicating its flexibility. Here we combined the AID system with an adaptation of CRISPR designed to increase tagging efficiency, in order to explore the biological roles of three apically localized CaMs in *T*. *gondii*.

## Materials and methods

### *Toxoplasma gondii* cultivation

The previously described RH*Δku80Δhxgprt* line [40], referred to as ku80^KO^, was used as a parent for the transgenic lines reported here. *Toxoplasma gondii* lines (S1 Table) were grown in confluent monolayers of human foreskin fibroblasts (HFF), obtained from the Boothroyd laboratory at Stanford University, and harvested as described previously [41]. Parasite cultures were determined to be mycoplasma negative using the e-Myco plus kit (Intron Biotechnology).

### Antibodies and chemicals

Primary antibodies used include: mouse anti-Ty (mAb BB2 [42]), rabbit HA (71–5500, ThermoFisher Scientific), mouse anti-HA (anti-HA.11, clone 16B12, Biolegend), rat anti-HA (Roche), rat anti-Flag (Biolegend), mouse mAb 45–15 anti-IMC1 (obtained from Gary Ward) [43], rabbit anti-GAP45 (obtained from Dominique Soldati-Favre) [5], mouse anti-c-myc (mAb 9E10, Life Technologies), rabbit anti-aldolase [44], rabbit anti-ROP5 [45], mouse mAb 6D10 anti-MIC2 [46], rabbit anti-GRA7 [47], rabbit anti-RON4 (obtained from John Boothroyd) [48] mouse mAb DG52 anti-SAG1 [49], mouse anti-ROP1 (mAb Tg49) [50], rabbit anti-MLC1 [51]. Indoleacetic acid (IAA, auxin), D-biotin and calcium ionophore A23187 were obtained from Sigma-Aldrich. Secondary antibodies used for immunofluorescence staining consisted of goat anti-mouse IgG or goat anti-rabbit IgG or goat anti-rat IgG conjugated to Alexa Fluor-488, Alexa Fluor-594 or Alexa Fluor-350 (Life Technologies). For Western blotting, secondary antibodies consisted of goat anti-mouse IgG or goat anti-rabbit IgG or goat anti-rat IgG conjugated to LiCor C800 or C680 IR-dyes and detected with an Odyssey Infrared Imaging System (LI-COR Biotechnology).

### Generation of CRISPR/Cas9 plasmids

A detailed description of plasmid design and construction is found in the supplementary materials and a list of plasmids generated (S2 Table) and primers used (S3 Table) are found in the supplemental materials. Plasmids for epitope tagging were generated using a common core cassette containing the *HXGPRT* selectable marker flanked by LoxP sites and placed downstream of a series of epitope tags (S1 Fig). To generate clean knockouts of specific genes, we used a previously described double sgRNA strategy to create separate double strand break at the 5’ and 3’ ends of a gene of interest, followed by selection for insertion of the PyrR-DHFR selectable maker flanked by LoxP sites and short, gene-specific homology regions [41]. Where necessary, we removed the selectable marker by transfection of the plasmid pmin-Cre-GFP [52] (obtained from Ke Hu) prior to disruption of a second gene or complementation. Complementation plasmids were generated in a plasmid containing the PyrR-DHFR selectable marker followed by a multiple cloning site containing 2Ty tag at the C terminus. Mutations were generated using Q5 mutagenesis (New England Biolabs), and plasmids verified by Sanger sequencing.

### Transgenic parasites

Freshly prepared tachyzoites were transfected by electroporation in CytoMix buffer, as previously described [53]. Transgenic parasites were selected following DNA transfection with the appropriate antibiotics including chloramphenicol (Cm) (20 μM), mycophenolic acid (MPA) (25 μg/ml), 6-xanthine (6Xa) (50 μg/ml), 6-thioxanthine (6TX)(50 μg/ml), or pyrimethamine (Pyr) (3 μM), and stable clones were isolated by limiting dilution on HFF monolayers grown in 96 well plates. Genotype information for clonal lines generated is listed in (S1 Table).

### TIR1 and YFP-AID plasmids and parasite lines

We adopted a previously described system for auxin-induced degradation [39] by expression of the *Oryza sativa* Transport Inhibitor Response 1 (TIR1) protein that was driven by the *TUB1* promoter and FLAG-tagged as a stable transgene in the ku80^KO^ line of *T*. *gondii* (S1 Table). To generate degradable fusions, the *Arabidopsis thaliana* AID coding sequence [39] was cloned into the HXGPRT tagging plasmid described above (S1 Fig, S2 Table). Details of the construction of these lines are provided separately [54]. We generated YFP-AID-3HA and CaM-AID-3HA lines in the TIR1 parental line by endogenous tagging using amplicons from the pLinker-AID-3HA-HXGPRT-LoxP tagging plasmid (S2 Table) and CRISPR/Cas9-mediated targeting (S1 Table).

### Cell fractionation and Western blotting

Freshly egressed parasites were re-suspended in PBS containing 1% Triton X-100 with either 5 mM EDTA or 5 mM CaCl_2_ in a 4°C ice bath, and centrifuged at 22,000*g* for 20 min. The supernatants were mixed with 5x Laemmli sample buffer. The pellets were washed in PBS, centrifuged again, and resuspended in PBS and 5x Laemmli sample buffer. The supernatant and pellet fractions were resolved by SDS-PAGE. Parasite lysates, or cell fractions, were processed for Western blotting using primary antibodies and Licor conjugated secondary antibodies, as described previously [41].

### Auxin induced degradation

To monitor the dose-dependent degradation of AID-tagged proteins, parasites grown in HFF monolayers were cultured with different doses of auxin, or with ethanol alone (0.1%), for 4 hr at 37°C. To monitor the time-dependence of AID degradation, parasites grown in HFF monolayers were treated with auxin (500 μM), or ethanol alone (0.1%), for different time intervals at 37°C. Following treatment, parasites were harvested and analyzed by Western blotting, as described above. Each experiment was performed at least three times with similar results and representative blots are shown.

### Indirect immunofluorescence (IFA) staining

Parasites cultured in HFF monolayers on coverslips were fixed in 4% formaldehyde in PBS, and permeabilized in 0.25% Triton X-100 (unless otherwise indicated) in PBS and blocked in 10% goat serum in PBS. Monolayers were incubated with different primary antibody combinations and visualized with secondary antibodies conjugated to Alexa Fluors. Images were captured using a 100x-oil objective (Ph3, N.A. 1.4) on an Axioskop 2 MOT Plus Wide Field Fluorescence Microscope equipped with an AxioCam and Axiovision LE64 software (Carl Zeiss, Inc.).

### Super-resolution microscopy

Fleshly lysed parasites were collected and re-suspended in PBS containing 3 μM A23187 to induce conoid extrusion and immediately added to coverslips coated with poly-L-lysine and incubated for 10 mins. The coverslips were washed with PBS and fixed with 4% formaldehyde in PBS, and then used for IFA with primary antibodies followed with secondary antibodies conjugated to Alexa Fluors. Images were acquired with a Zeiss 63x/1.4 NA objective using Airyscan super-resolution module on a Zeiss LSM 880 confocal microscope. Images were processed and prepared using ZEN Black software, and at least 15 images were analyzed for each of the lines.

### Growth and replication assays

Growth of control (ethanol alone (0.1%) or auxin (500 **μM**) treated parasites on HFF monolayers was monitored by plaque formation after 6–8 days of culture, as described previously [41]. Replication was monitored based on the average number of parasite / vacuole at 24 hr, as described previously [41]. Each experiment included triplicate coverslips and three independent experiments were performed. **I**mages were taken with Axiocam 503 color in a Zeiss Axio Observer D.1 microscope at 2.5x magnification, and plaque sizes were measured with Zeiss software Zen 2.3 Pro.

### Evacuole formation

Parasites were treated with 1μM cytochalasin D for 10 min, and then used to challenge HFF monolayer for 30 min in media containing 1μM cytochalasin D. Samples were fixed with 4% formaldehyde in PBS and stained with antibodies against ROP1 (mAb Tg49) and GAP45, followed by Alexa-conjugated secondaries. Parasites (as detected by GAP45 staining) with and without evacuole vacuoles (as detected by ROP1 staining) were counted from at least 100 parasites per sample. Data were plotted as the ratio of parasites that were associated with a cluster of evacuoles. Three independent experiments were performed in triplicate.

### Parasite invasion

Parasite invasion was monitored using a two-color IFA assay to discriminate extracellular from intracellular parasites, as described previously [11]. Following growth for 40–44 hr in 500 **μM** auxin, or ethanol alone (0.1%) parasites were harvested and used to challenge HFF cells grown on coverslips in 24-well plates for 20 min. Extracellular parasites were labeled with mAb DG52, and following saponin permeabilization, all parasites where stained with rabbit anti-GAP45 followed by secondary antibodies labeled with Alexa Fluors. For pulse-invasion, control or auxin treated parasites were used to challenge HFF monolayers at high multiplicity in a short pulse-invasion (i.e. for 3 min), as described previously [17]. Monolayers were processed for IFA and strained with rabbit anti-RON4 antibody, and mouse mAb DG52 to SAG1, without permeabilization, followed by secondary antibodies conjugated to Alexa Fluors. At least 150 parasites on each coverslip with triplicates per experiment were scored and the experiment was repeated three times.

### Parasite egress

Parasites grown in HFF monolayers for 30 hr with 500 μM auxin, or ethanol alone were treated with either 3 μM A23187 (%w/v DMSO) or DMSO alone for different time intervals at 37°C. Following incubation, samples were stained by IFA using antibodies against IMC1 (mouse) and GRA7 (rabbit) and followed by secondary antibodies conjugated to Alexa Fluors. Samples were examined by epifluorescence microscopy and the percentages of egressed (i.e. dispersed parasites with a remnant GRA7 positive vacuole) vs. intact vacuoles (i.e. parasites remained clustered within a vacuole defined by the outline of GRA7 staining) were determined from ≥ 200 vacuoles per coverslip. Each experiment was done in triplicate and three independent experiments were performed.

### Conoid extrusion

Parasites grown in HFF cells with 500 μM auxin, or ethanol alone for 40–44 hr were harvested, re-suspended in PBS containing either 3 μM A23187, or DMSO alone, and added to poly-lysine coated coverslips for 5 min at 37°C. Adherent parasites were fixed with 4% formaldehyde in PBS, rinsed in PBS, mounted on glass slides, and examined on an Axioskop 2 MOT Plus Wide Field Fluorescence Microscope (Carl Zeiss, Inc.). At least 150 parasites were counted for each of three coverslips from three independent experiments and values were expressed as ratios of total parasites.

### Microneme secretion

Microneme secretion was assayed using a quantitative secretion assay that relies on a Glaussia luciferase (Gluc) fusion with MIC2, as described previously [55]. In brief, the parental and AID fusion lines were transfected with a plasmid expressing MIC2-GLuc-c-myc [55], and stable clones selected with MPA/Xa. MIC2-Gluc-myc expressing lines were grown with 500 μM auxin, or ethanol alone for 40–44 hr, mechanically egressed and stimulated for secretion using 1% BSA (Sigma) and 1% ethanol at 37°C for 10 min, and luciferase activity measured as described previously [55]. The experiment was repeated three separate times with three internal technical replicates.

### Parasite motility

Parasites were treated with 500 μM auxin or ethanol during 40–44 hours of growth in HFF monolayers and then harvested by mechanical lysis. Gliding motility was performed in Ringer's media and 3% FBS and captured by time-lapse microscopy, as described previously (22). Conventional bright-field images were captured at ~10 frame/ sec using a 63x Oil Plan Apochromat Lens (N.A. 1.4). Three independent experiments including at least 20 parasites per condition were used to score gliding behaviors (i.e. circular, helical, or twirling) or not productively moving.

### Analysis of biotinylated proteins

BirA-tagged lines were used to infect HFF monolayers on coverslips in a 24-well plate, and grown for 24 hr with addition of 180 μM D-Biotin in D10 at 37°C with 5% CO_2_. Monolayers were processed for IFA and stained with rabbit anti-HA antibodies followed by secondary rabbit antibodies conjugated with Alexa Fluor-594, and streptavidin Alexa Fluor-488 (Life Technologies). Biotinylated proteins were purified as described previously [56, 57], except that parasites were grown in media containing 180 μM D-biotin for 20 hr, lysed in the following buffer (10 mM Tris-HCl, pH7.4, 100 mM NaCl, 1 mM EDTA, 1 mM EGTA, 1% Triton X-100, 10% glycerol, 0.2% SDS, 0.5% deoxycholate, supplemented with protease inhibitors (Roche), and sonicated using a microtip in 550 sonic dismembrator (Fisher Scientific).

### Mass spectrometry analysis

Streptavidin beads containing biotinylated proteins were dissolved in 0.1 M ammonium bicarbonate and then reduced with DTT and alkylated with 10 mM iodoacetamide. Trypsin was then added and digestion was carried out overnight at 37°C. Mass spectrometric analysis was carried out on a nanoLC-MS/MS using a 2 hr gradient on a 0.075 mmx250mm C18 Waters CSH column feeding into a Q-Exactive HF mass spectrometer. Data were then analyzed with Mascot and Scaffold and compared to the *T*. *gondii* annotated proteome (http://ToxoDB.org, release 29). Proteins were identified based on the criteria of two or more peptides matching with > 95% confidence in two replicate experiments. Normalized spectral abundance factors (NSAFs) were determined by dividing the spectral abundance for each protein by its length (amino acid residues) and normalization against the sum of all SAFs (after removing redundant proteins), as previously described [58].

### Molecular modeling

Comparative models were built from template X-ray crystal structures from the protein database (PDB) files that were identified by searching and aligned using HHSEARCH/HHpred [59], RaptorX [60], and Sparks-X [61] as implemented on the Robetta server (robetta.org<http://robetta.org>). Alignments were clustered and comparative models were generated using the RosettaCM protocol [62]. Mutations were performed with UCSF Chimera [63] using the Dunbrack rotamers library [64]. Calcium binding sites were modeled by adjusting torsion angles of D/E side-chains to equivalent positions relative to the template PDB:2N6A_A, followed by rounds of energy minimization using steepest descent (30 steps, step size 0.02A) until no van der Waals overlap ≥ 0.6A was detected.

### Statistical analyses

For data that were normally distributed, differences in the means were assessed by one-way or two-way ANOVA with Tukey’s multiple comparison. For data that were either not normally distributed, or were sample sizes were too small to validate the distribution, sample distributions were compared using Mann Whitney or Kruskal-Wallis non-parametric tests to compare selected pairs of column with Dunns’ multiple comparison. In both cases, *P* ≤ 0.05 was considered significant.

## Results

### CRISPR tagging and essentiality of CaMs

**Fig 1:**
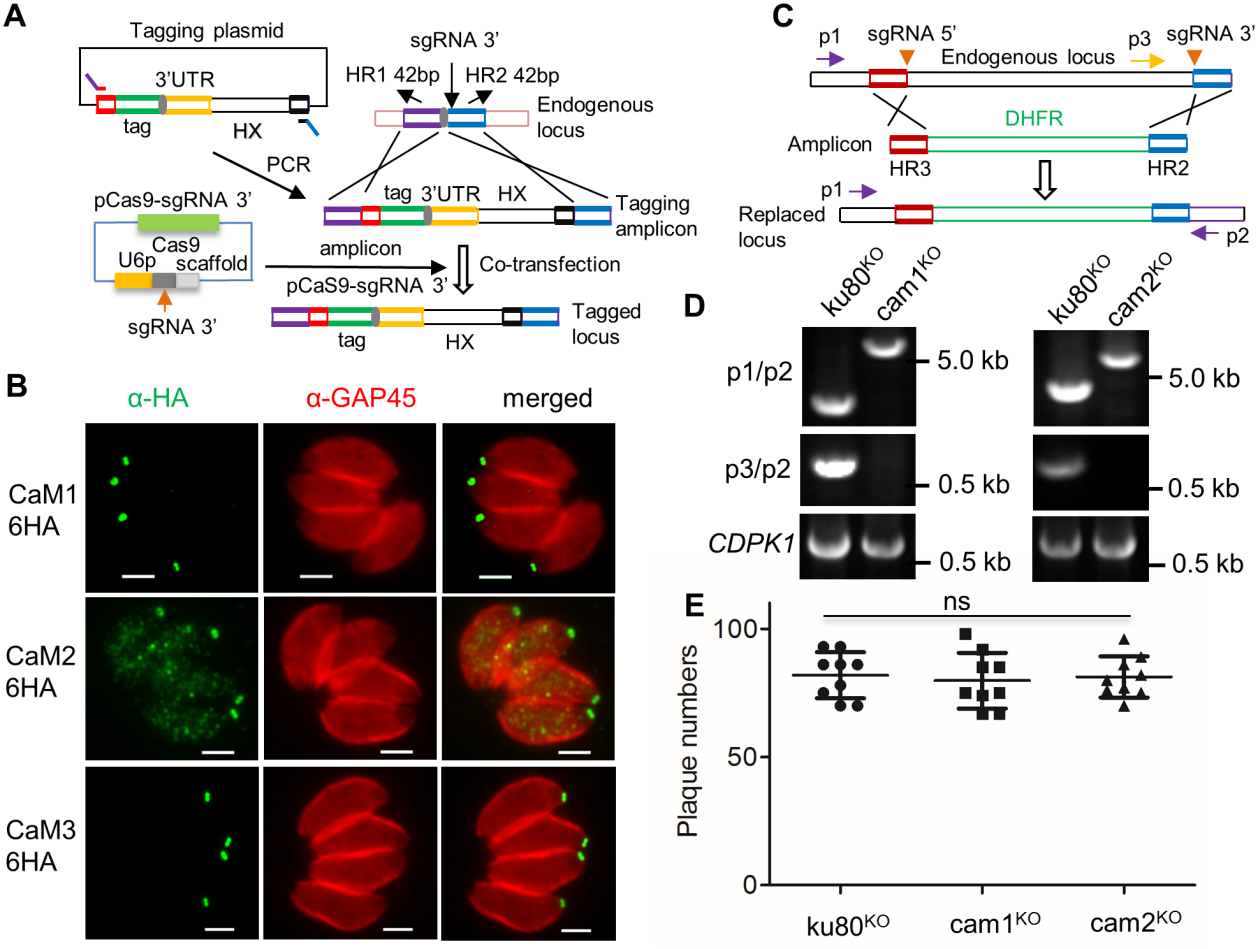
Endogenous tagging and generation of knockouts in *T*. *gondii*. **A**. Schematic of the CRISPR/Cas9 tagging system. Tagging plasmids were generated with various tags (green box) flanked by common ends (red and black boxes) and including a common stop codon (gray box) followed by the *HXGPRT* 3’ UTR (yellow box) and the selectable marker HXGPRT. Amplification of this central region with primers that contained short homology regions HR1 (purple box) and HR2 (blue box) together with the common flanks (red and black boxes) generated products for gene-specific tagging. Co-transfection of these amplicons with a CRISPR/Cas9 plasmid bearing the gene-specific single guide RNA (sgRNA3’) was used to add an epitope tag (green box) at the C-terminus of the endogenous locus. See S1 Fig for more details. **B**. Localization of CaM1, CaM2 and CaM3 containing C-terminal 6HA tags. Detected with mouse anti-HA (green) and rabbit anti-GAP45 (red). Scale bar, 2 μM. **C**. Schematic of the double CRISPR/Cas9 gRNA system used for generation of clean knockouts using two sgRNAs matching the 5’ and 3’ ends of the coding sequence. The entire coding sequence was replaced by the DHFR marker flanked by short homology regions (HR3, red; HR2, blue). Primers (p) used for diagnostic PCR. **D**. Diagnostic PCR of knockouts compared to the parental ku80^KO^ line. *CDPK1*, PCR control. **E.** Plaque numbers formed by the knockouts compared to the parental ku80^KO^ line. ns, not significant, analyzed by one-way ANOVA.

To develop an efficient system for endogenously tagging the C-termini of candidate genes, we used site-specific CRISPR/Cas9 cleavage to increase the efficiency of homologous integration (Fig 1A and S1A Fig). CRISPR/Cas9-sgRNA plasmids were designed to target sgRNA close to the stop codon (illustrated in S1B and S1C Fig). Tagging was accomplished by electroporation of the CRISPR Cas9-sgRNA plasmid together with amplicons generated from plasmids containing an epitope tag followed by a selectable marker cassette (S1A and S1D Fig). Tagging amplicons incorporated primers containing short homology regions (~40bp) to the gene of interest (illustrated in S1B Fig). Introduction of these amplicons, together with the CRISPR/Cas9-sgRNA plasmid into the ku80^KO^ line, which is deficient in non-homologous end joining, assured high fidelity, site-specific integration at the endogenous locus on the chromosome. To allow for reuse of the marker, the resistance cassette was flanked by LoxP sites (S1E Fig), which were removed by transfection of pMinCre, as described previously [52]. This system offers the advantages of high efficiency cleavage by CRISPR/Cas9-sgRNA to drive integration at the site of interest, combined with a common pair of primers that can be used to generate amplicons containing a variety of different tags, which do not require separate cloning steps (S1A Fig). The system was then applied to examine the localization, essential function, and binding interactions of three apically localized CaM like proteins in *T*. *gondii*.

CRISPR/Cas9-sgRNA plasmids designed to target near the 3’ end of *CaM* genes were generated with Q5 DNA mutagenesis (S1C Fig), using guidelines described previously [31, 65] and co-transfected with their corresponding 6HA tag amplicons containing 42 bp homology regions matching the gene of interest (Fig 1A). Resistant clones were isolated, confirmed by PCR, and tested by immunofluorescence assay (IFA). CaM1 and CaM2 were localized to the apical end of the cell, extending beyond the inner membrane complex that was detected by GAP45, as described previously [16] (Fig 1B). A similar distribution was observed for CaM3 (Fig 1B). To test for essentiality, we used two sgRNAs targeting the 5’ and 3’ of the coding sequence to delete the entire coding sequence [41, 54] (Fig 1C). This strategy is similar in that it pairs a drug resistance cassette flanked by 42 bp homology regions with the gene-specific CRISPR/Cas9 double sgRNA plasmid (Fig 1C). We were able to easily generate cam1^KO^ and cam2^KO^ clones as shown using diagnostic PCR (Fig 1C and 1D). The cam1^KO^ and cam2^KO^ mutants formed normal numbers of plaques of equal size to the parental ku80^KO^ line (Fig 1E), indicating they are not essential for growth *in vitro*. However, we were unable to obtain a cam3^KO^ clone, suggesting that it might be essential. We also attempted to sequentially delete *CaM2* in the cam1^KO^ line after excision of the resistance marker. We were unable to isolate double mutants using this strategy, or the reciprocal approach, suggesting the combined loss of these genes was deleterious.

#### Development of an auxin-inducible degron (AID) system for *T*. *gondii*

**Fig 2:**
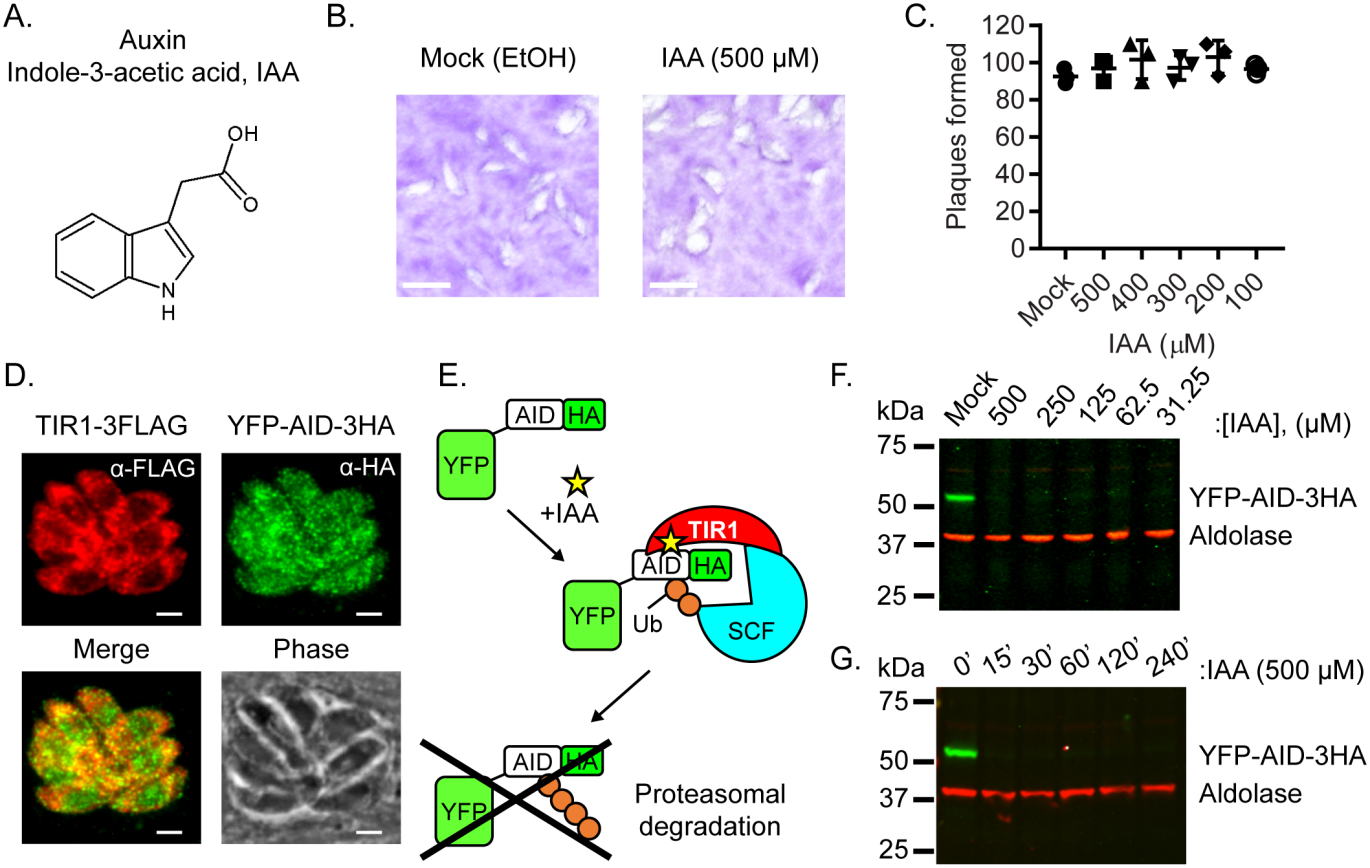
Development of the auxin inducible degron (AID) system in *T*. *gondii*. **A.** Structure of auxin indole-3-acetic acid (IAA). **B**. Plaque formation of the ku80^KO^ line grown in 500 μM IAA or ethanol control (0.1%) (mock) for 6 days. Scale bar = 2 mm. **C.** Plaque formation of *the* ku80^KO^ line grown in IAA or ethanol control (0.1%) mock) for 6 days. Mean ±S.D. from three independent experiments with triplicates for each (n = 9). ns, not significant, analyzed by one-way ANOVA. **D.** Heterologous co-expression of *Oryza savita* auxin receptor TIR1 (TIR1-3xFLAG)(parental line) (red) and an N-terminal YFP fusion with the *Arabidopsis thaliana* IAA17 (AID) (YFP-AID-3xHA) (green) in the TIR1 background. Scale bar, 2 μm. **E.** Schematic illustration of conditional degradation of AID-tagged proteins in *T*. *gondii*. **F.** Dose-response and **G.** time course of YFP-AID-3HA knockdown following IAA treatment by Western blotting with antibodies to YFP-AID-3HA (mouse anti-HA) or Aldolase (as a loading control).

To further explore the role of essential genes, or synthetic lethal interactions, we adapted a plant auxin-induced degradation system for use in *T*. *gondii*. We first established that addition of the plant hormone indoleacetic acid (IAA or auxin) did not alter the growth of the parental ku80^KO^ line at concentrations up to 500 μM (Fig 2A, 2B and 2C). Expression of a codon-optimized *TIR1* gene from rice resulted in cytosolic localization of the TIR1 protein in *T*. *gondii* (Fig 2D). To test the efficiency of protein degradation in this system, we expressed YFP as a fusion with AID-3HA in the TIR1 parental strain (Fig 2D and 2E) (S1 Table). Addition of auxin rapidly degraded the YFP-AID-3HA protein across a range of different concentrations (Fig 2F), and loss of the protein occurred within 15 min at an auxin concentration of 500 μM (Fig 2G).

**Fig 3:**
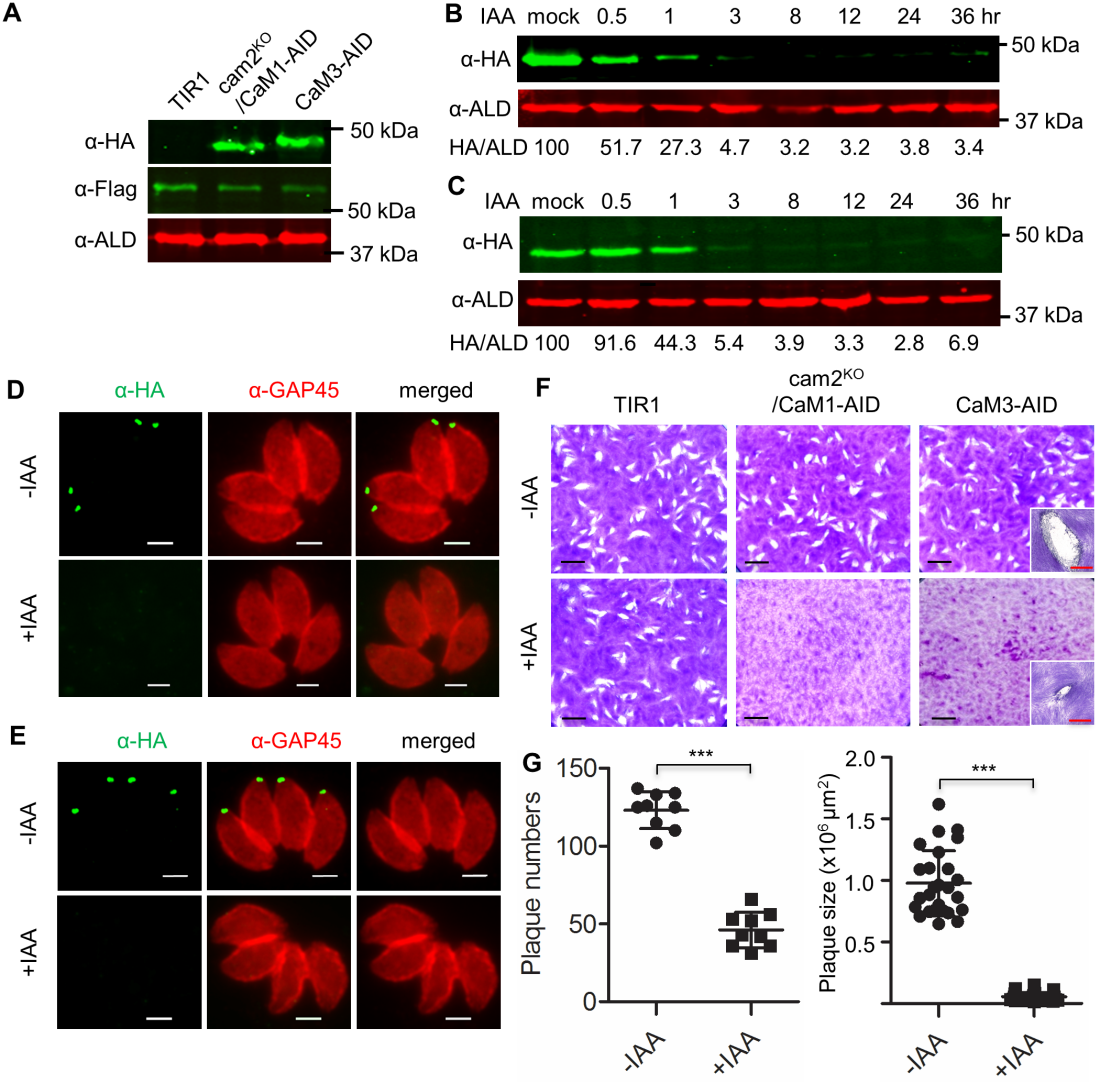
Generation of AID tagged lines in the TIR parental line of *T*. *gondii*. **A.** Western blot analysis using antibodies to detect CaM1-AID or CaM3-AID (mouse anti-HA to the AID-3HA tag), TIR1-3Flag (rat anti-Flag) and aldolase (rabbit anti-aldolase, ALD). **B and C.** Degradation of AID tagged proteins in cam2^KO^*/*CaM1-AID (**B**) and CaM3-AID (**C**) lines after addition of auxin (500 μM IAA) for different time periods. Mock indicates parasites grown with 0.1% ethanol for 36 hr. CaM1-AID or CaM3-AID proteins were detected with mouse anti-HA and rabbit anti-aldolase (ALD) antibodies served as a loading control. Band intensities were analyzed by ImageJ, and ratios of anti-HA vs. anti-ALD signal were calculated (HA/ALD) and expressed as a percentage of the mock treatment (i.e. 100%). **D and E**. Degradation of AID tagged proteins in cam2^KO^*/*CaM1-AID (D) and CaM3-AID (E) parasites after 24 hr incubation with 500 μM IAA (+IAA) or ethanol vehicle 0.1% (-IAA). CaM1-AID or CaM3-AID proteins were detected with mouse anti-HA (green) and rabbit GAP45 (red) antibodies served as a control to label the parasite. Scale bar, 2 μM. **F**. Plaque formation by parasites grown on HFF monolayers. Scale bar, 0.5 cm. Insert images in the CaM3-AID line, scale bar (red) = 1 mm. **G**. Measurement of plaque numbers and sizes for the CaM3-AID line treated with and without auxin. N≥ 25, ***, *P* < 0.0001. Mann Whitney non-parametric test.

To examine the role(s) of essential genes, the double CRISPR sgRNA strategy was first applied to delete *CaM2* in the TIR1 parental strain (S2A Fig), and then *CaM1* was tagged with AID-3HA, yielding the cam2^KO^/CaM1-AID clone (S2A Fig) (S1 Table). A similar strategy was used to generate the cam1^KO^/CaM2-AID clone (S2C Fig) (S1 Table). Additionally, a CaM3-AID clone was generated in a single step by transfection of a CRISPR Cas9-sgRNA3’ plasmid with an AID-3HA tagging amplicon (S2B Fig) (S1 Table). The correct genetic alterations were confirmed by PCR (S2A–S2C Fig) and protein expression was verified by Western blotting with antibodies against HA (detecting the AID fusion), and FLAG (detecting the TIR1 protein) (Fig 3A). Addition of auxin led to rapid degradation of CaM-AID fusion proteins as shown by Western blot (Fig 3B and 3C, S2D Fig) and IFA (Fig 3D and 3E). Interestingly, the kinetics and extent of degradation for the CaM1-AID (Fig 3B) and CaM3-AID (Fig 3C) proteins was somewhat slower than that of YFP-AID (Fig 2F). Substantially less protein was present after 1 hr of auxin treatment, and nearly complete shutdown of the CaM proteins was observed by ≥ 3 hr (Fig 3B and 3C). Despite this efficient shut down, a trace of protein remains detectable by Western Blot, even out to 36 hr (Fig 3B and 3C). The TIR1 parental line was able to form plaques when grown in the presence or absence of auxin (Fig 3F). The cam2^KO^/CaM1-AID (Fig 3F) and cam1^KO^/CaM2-AID (S2E Fig) lines were normal when grown in the absence of auxin, but they were unable to form plaques with addition of auxin. Because these two lines have similar phenotype, the cam2^KO^/CaM1-AID line was chosen for further analysis, as described below. The CaM3-AID line was severely inhibited upon addition of auxin but still formed very small plaques, compared to normal growth without auxin (Fig 3F and 3G). In summary, the TIR1-AID system proved highly efficient for degrading target proteins and confirming essential or synthetic lethal phenotypes.

#### Involvement of CaMs in parasite replication, conoid extrusion, and secretion

**Fig 4:**
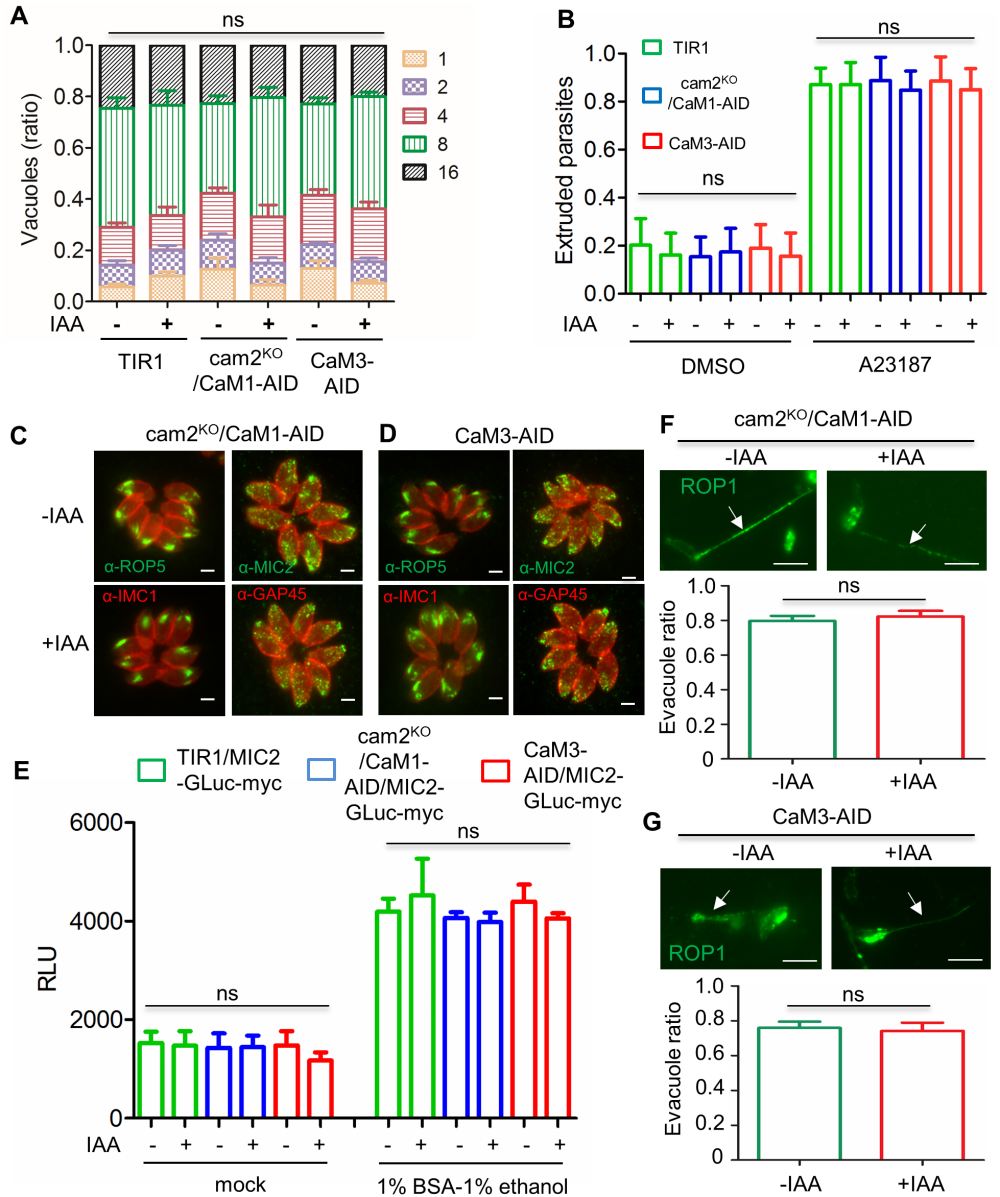
Analysis of parasite replication, conoid protrusion, apical organelle distribution and secretion in parental and mutant lines. **A**. Parasite replication after 24 hr incubation with ± IAA (500 μM vs 0.1% ethanol). ns, not significant. **B**. Proportion of parasites with extruded conoid. Parasites grown for 2 days ± IAA (500 μM vs 0.1% ethanol), stimulated with 3 μM A23187 or DMSO vehicle control for 10 min. ns, not significant. **C and D**. Distribution of MIC2 (mouse anti-MIC2 (green) and ROP5 (rabbit annti-ROP5 (green) upon depletion of AID fusion proteins. Parasites grown ± IAA (500 μM vs 0.1% ethanol) for 24 hr in HFF monolayers and stained for IFA. Parasites were counterstained with mouse anti-IMC1 (red) or rabbit anti-GAP45 antibodies (green). Scale bar, 2 μM. **E.** Quantification of micronemal secretion using MIC2-GLuc-myc reporter lines. Parasites were grown for 2 days ±IAA (500 μM vs 0.1% ethanol), stimulated with 1% ethanol—1% BSA and secretion was monitored by releases of luciferase (see methods). Relative Luminescence Unit (RLU)**.** ns, not significant. **F and G.** Detection of rhoptry secretion by ROP1 staining. Parasites were grown for 2 days ± IAA (500 μM vs 0.1% ethanol), harvested and used to detect formation of evacuoles (arrows) on fresh monolayers of HFF cells in the presence of cytochalasin. Parasites were counted from triplicate samples on three separate experiments and ratios of parasites associated with evacuoles in were plotted. Scale bar, 5 μm. Panels **A**, **B**, **E**, **F, G** mean ± S.D. from three independent experiments with triplicates for each (n = 9). One-way ANOVA with Tukey’s multiple comparison test for **B** and **E** and two-way ANOVA with Tukey’s multiple comparison test for pair-wise multiple comparisons across each vacuole size for **A**, Man-Whitney non-parametric test for **F** and **G**.

To pinpoint the defect(s) of cells following degradation in the AID fusion lines, a series of phenotypic assays were performed to test the role of CaMs in the lytic cycle. We compared the response of the cam2^ko^/CaM1-AID clone and the CaM3-AID clone to the TIR1 parental line when grown in the presence *vs*. absence of auxin. Following degradation of CaMs by addition of auxin, there was no defect in intracellular replication (Fig 4A), conoid extrusion in response to ionophore (Fig 4B), or distribution of apical organelles revealed by staining for MIC2 or ROP5 (Fig 4C and 4D). Microneme secretion in response to BSA-ethanol, potent agonists for secretion [55], was also normal following degradation of CaMs by growth in auxin (Fig 4E). Finally, formation of evacuoles as measured by secretion of rhoptry proteins after treatment with cytochalasin D [66], was normal following degradation of CaMs by growth in auxin (Fig 4F and 4G). Consequently, we concluded that the severe defect on the lytic cycle must lie in other important biological processes.

#### Involvement of CaMs in parasite egress and invasion

**Fig 5:**
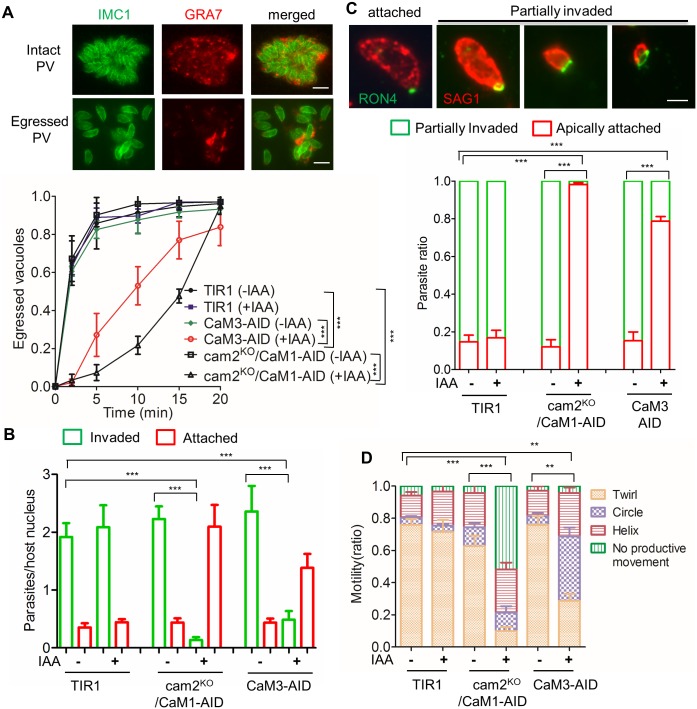
Analysis of egress, invasion, and motility in parental and mutant lines. **A**. Parasites grown for 30 hr ± IAA (500 μM vs 0.1% ethanol) were stimulated with 3 μM A23187 to simulate egress. Rabbit anti-GRA7 (red) and mouse anti-IMC1 (green) antibodies were used to distinguish intact vs. egressed vacuoles. *** *P ≤ 0*.*0001*, significant for the time points of 2, 5, 10 and 15 min, but not significant for 0 and 20 min. Scale bar, 5 μM. **B**. Quantitative analysis of invasion by parasites grown for 2 days ± IAA (500 μM vs 0.1% ethanol) and used to challenge fresh HFF monolayers on coverslips for 20 min. Extracellular parasites (invaded) were distinguished from those that remained extracellular (attached) by differential IFA staining (see methods). *** *P* ≤ 0.0001. **C.** Evaluation of cell entry past the moving junction. Parasites grown for 2 days ± IAA (500 μM vs 0.1% ethanol) were used to challenge fresh HFF monolayers on coverslips for 3 min, fixed and stained with rabbit anti-RON4 (green) and mouse anti-SAG1 (red) without permeabilization. Parasites with RON4 dots were considered to be apically attached (red column), and parasites with RON4 positive rings were classified as partially invaded (green column). *** *P ≤ 0*.*0001*. Scale bar, 2 μM. **D**. Parasite motility as monitored by video microscopy. Parasites grown for 2 days ± IAA (500 μM vs 0.1% ethanol) were allowed to glide on serum-coated coverslips. Time-lapse video microscopy was used to score different motile behaviors. *** *P* ≤ 0.0001, the cam2^KO^CaM1-AID line showed significant decrease in twirling and increase non-productive movement when grown in +IAA *vs*. -IAA or the TIR1 parental line, **, *P* ≤ 0.0001, the CaM3-AID line showed a significant decrease in twirling and increase in circling when grown in +IAA *vs*. -IAA or the TIR1 parental line. Panels **A**, **B**, **C**, **D** represent means ± S.D. from three independent experiments with triplicates for each (n = 9). Two-way ANOVA with Tukey’s multiple comparison test for **A**, **C** and **D**, and one-way ANOVA with Tukey’s multiple comparison test for **B**.

The plaque assay where CaM-depleted mutants showed a striking phenotype requires multiple rounds of infection and hence relies on parasite motility, invasion, and egress. To explore these phenotypes more directly, we utilized separate assays that assess each of these steps individually. When CaM proteins were degraded by growth in auxin, both the cam2^KO^CaM1-AID and CaM3-AID strains showed a strong egress delay compared to the TIR1 parental line (Fig 5A). Likewise, the cam2^KO^/CaM1-AID and the CaM3-AID lines showed a significant decrease in cell invasion, and corresponding increase in attachment, when grown in auxin, compared to the TIR1 parental line (Fig 5B). To define the point at which attached, but non-invaded proteins were blocked, we used a short pulse-invasion assay and stained parasites during invasion with RON4 to define the moving junction [48], and SAG1 to reveal the portion of the parasite that was extracellular (done without detergent). The majority of parasites in the TIR1 parental line, and the AID strains grown without auxin, were able to form a RON4 ring and move partially into the cell, while a minority remained apically attached but did not move past the junction (Fig 5C). In contrast, nearly all of cam2^KO^/CaM1-AID parasites and 80% of CaM3-AID parasites grown in auxin were apically attached but unable to move past the junction (Fig 5C). Since migration past the junction requires actin-myosin based motility [11, 12], we assessed whether parasites were impaired in gliding motility. Time-lapse video microcopy revealed that cam2^KO^/CaM1-AID and CaM3 lines grown in auxin showed severe defects in motility on 2-D substrates (Fig 5D). The cam2^KO^/CaM1-AID clone showed an increase in non-motile cells (no productive movement) and a decrease in twirling, while the CaM3-AID line showed a decrease in twirling and an increase in circular gliding, which does not lead to cell invasion (Fig 5D). Hence, it is likely that the strong defect in plaque formation observed when the cam2^KO^/CaM1-AID and CaM3 lines were grown in auxin, resulted from defects in parasite egress, invasion, and motility.

#### Role of EF hands in CaM structure and function

**Fig 6:**
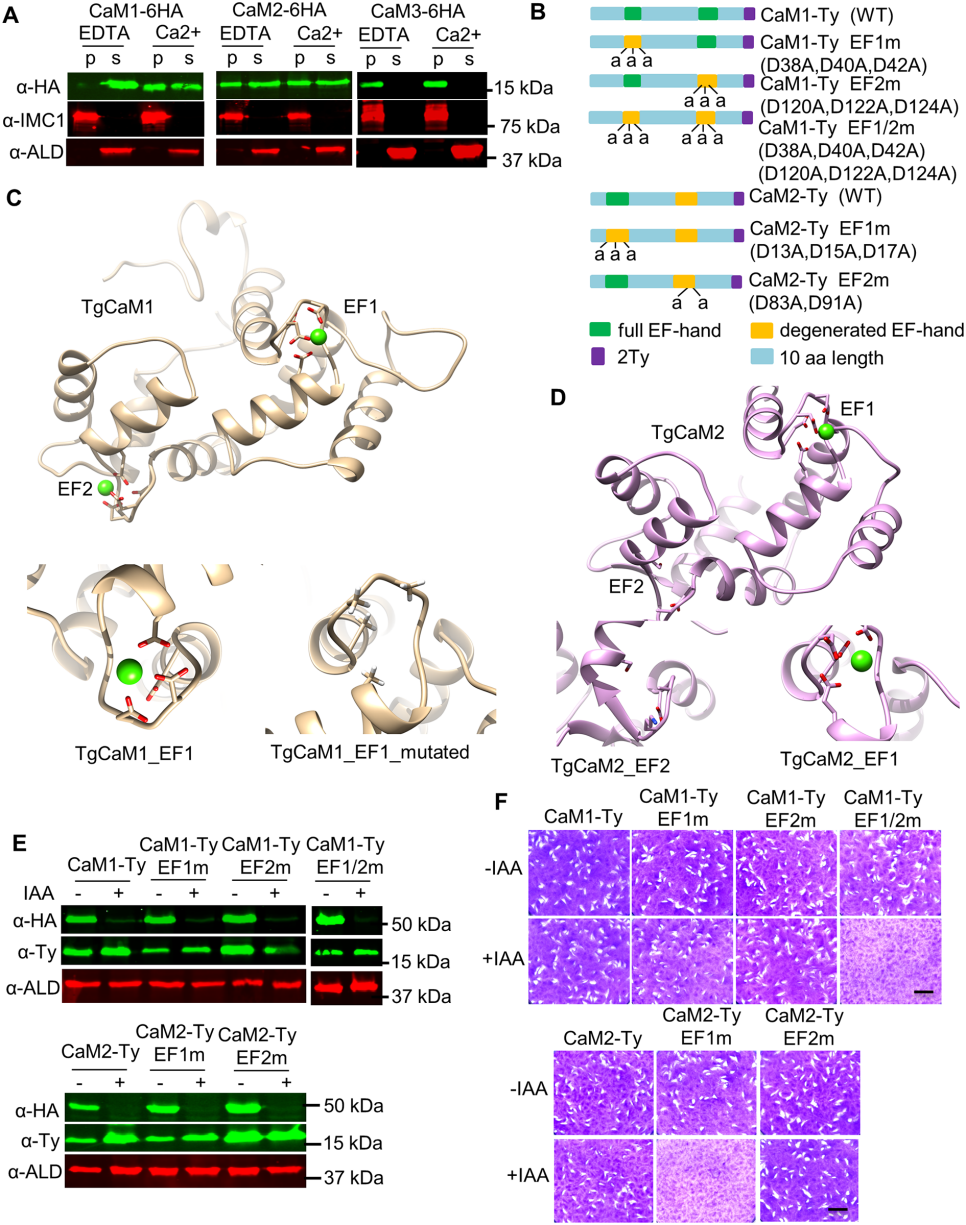
Assessment of the roles of EF hand domains in CaM1 and CaM2. **A**. Calcium-dependent solubility as detected by cell fractionation and Western blotting. Tagged parasites were lysed in 1% Triton X-100 in the presence of either 5 mM EDTA or 5 mM CaCl_2_ and fractionated by centrifugation. CaM1, CaM2, or CaM3 were detected with mouse anti-HA (green), while mouse anti-IMC1 was used as a control for the pellet (p) and rabbit anti-aldolase (ALD) was used as a control for the supernatant (s). **B**. Diagram of wild type (WT) and CaM1 and CaM2 mutants showing the residues in conserved or degenerated EF hands (predicted by ScanProsite). Names of the proteins are shown to the right (i.e. D38A represents an Asp residue at D38 that was mutated to Ala). **C and D**. Structural modeling of TgCaM1 (**C**), TgCaM2 (**D**) highlighting their EF hand domains. **C**, Top: Structure of TgCaM1 is shown with conserved Asp residues (red) chelating a calcium iron (green ball). Bottom: enlargement of the TgCaM1 EF1 domain showing the wild type (left) and the triple EF1m mutant (right). **D**, Top: The structure of TgCaM2 is shown with conserved Asp residues (red) in the EF1 but not in EF2. Bottom: enlargement of TgCaM2-EF1 showing intact EF1 domain that chelate calcium (right) and the degenerate EF2 domain (left). **E**. Western blot detection of CaM mutants grown for 2 days ± IAA (500 μM vs 0.1% ethanol). Cell pellets were resolved by SDS-PAGE and Western blotted using mouse anti-HA to detect CaM-AID fusions, mouse anti-Ty to detect complementing alleles, and rabbit anti-aldolase (ALD) antibodies as a loading control. **F**. Evaluation of complementation by plaque formation. Scale bar, 0.5 cm.

Although all three CaMs studied here have predicted EF hand domains, they differ in the degree of conservation, which might be expected to influence their responsiveness to calcium. CaM1 and CaM2 contain one or more intact EF hand domains suggesting their functions may be regulated by calcium. To examine their calcium-dependence, we fractionated cells into soluble (cytoplasmic) and insoluble (cytoskeleton and large protein complexes) and assess the partitioning of the CaMs under conditions where calcium was in excess vs. when it was chelated by EGTA. Consistent with the presence of conserved EF hands, CaM1 was highly soluble in the absence of calcium, but was shifted to the pellet in the presence of calcium (Fig 6A). This result suggests that CaM1 is soluble when not bound to calcium and that it interacts with insoluble components (e.g. the cytoskeleton) in the presence of calcium. In contrast, CaM2 was equally partitioned in the supernatant and pellet in both the presence and absence of calcium, indicating its interaction with other partners may not be calcium dependent (Fig 6A). CaM3 contains two degenerate EF hands, suggesting it is not calcium-dependent. When subjected to cell fractionation, CaM3 was found exclusively in the pellet in both conditions (Fig 6A), suggesting it forms stable interactions with some component of the insoluble fraction (e.g. cytoskeleton). Interestingly, CaM1 has two conserved EF hands, while CaM2 has one conserved and one degenerate EF hand (Fig 6B). Homology modeling suggested that calcium binds in an Asp-rich pocket formed by the conserved EF hand domains 1 and 2 in CaM1 (Fig 6C). Although calcium was also modeled to bind into the conserved EF hand domain 1 in CaM2, it is not predicted to bind in the degenerate EF hand domain 2, which lacks conserved Asp residues (Fig 6D). To investigate the functional role of calcium binding, we introduced mutations in the EF hands of CaM1 and CaM2 to change conserved Asp to Ala (Fig 6B), changes that are predicted to ablate calcium binding (Fig 6C and 6D). The wild type and mutant versions were stably transfected into the cam2^KO^/CaM1-AID strain, and expression was tested in the absence and presence of auxin (Fig 6E). As expected the wild type copies of CaM1 and CaM2 were fully able to complement the cam2^KO^/CaM1-AID clone grown in the presence of auxin (Fig 6F). Somewhat surprising, mutation of either of the intact EF hands in CaM1 did not result in a defect, while mutation of both EF hands together resulted in failure to rescue the growth defect of the cam2^KO^/CaM1-AID clone grown in the presence of auxin (Fig 6F). Similarly, mutation of the remaining intact EF hand in CaM2 failed to rescue growth (Fig 6F). These findings are consistent with calcium binding to the conserved EF hands in regulating the functions of CaM1 and CaM2.

#### Association of CaMs with MyoH

**Fig 7:**
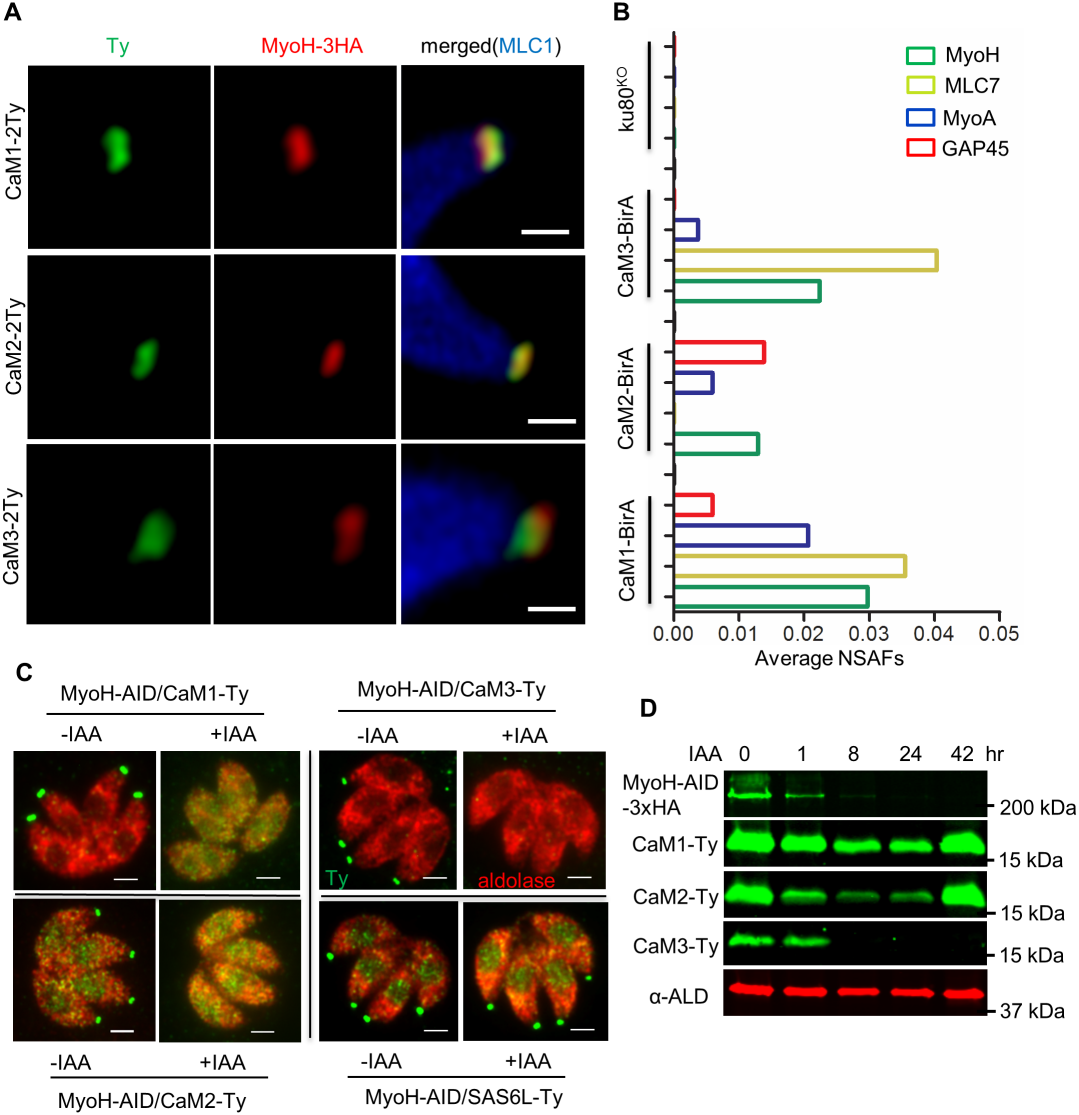
Interaction of CaM1, CaM2 and CaM3 with MyoH at the conoid. **A.** Localization of Ty-tagged CaMs (green) in a MyoH-3HA strain (red) by AiryScan super-resolution microscopy. Conoid extrusion was stimulated prior to fixation and IFA staining, MLC1 (blue) used to detect the parasite. Scale bar, 0.5 μm. **B.** Biotin labeling of CaM lines labeled with BirA and analyzed by MS/MS. Normalized Spectral Abundance Factors (NSAF) were plotted as average values from the combination of two independent experiments. Further data are found in S4 Table. **C.** Localization of CaMs upon depletion of MyoH. Parasites were grown for 8 hr ± IAA (500 μM vs 0.1% ethanol and evaluated by IFA using mouse anti-Ty (green) and rabbit anti-aldolase (red) antibodies. SAS6SL was tagged with Ty and served as a control. **D.** Western blot detection of CaMs (anti-Ty) in the MyoH-AID (anti-HA) strain. Rabbit aldolase (ALD) as a loading control. See S4 Fig for more details.

In comparing the functions affected by simultaneous loss of CaM1 and CaM2, or degradation of CaM3 alone, it was evident that these mutants phenocopied those previously described for MyoH [7] (Table 1). This similarity suggested that these CaM-like proteins may regulate the activity of MyoH, a specialized myosin that is localized to the conoid [7]. Consistent with this, all three CaMs were colocalized with MyoH in a narrow band at the tip of the protruded conoid, as shown by super-resolution microscopy (Fig 7A). Using the CRISPR-Cas9-mediated tagging technology we also constructed BirA fusions with the CaMs, and used these lines for biotinylation of proximal interacting proteins, as described previously [67] (S3A and S3B Fig). Biotinylation by the BirA-tagged CaM1, CaM2, or CaM3 fusions labeled the conoid, as shown by streptavidin-Alexa Fluor-488 staining (S3C Fig). These findings indicate that the BirA fusions are properly localized, and hence amenable to detecting interacting proteins in intact cells. Mass spectrometry of biotinylated and purified proteins revealed that CaM1-BirA, CaM2-BirA, and CaM3-BirA labeled a number of apically localized proteins including cytoskeletal components, DCX [68] and RNG2 [69], as well as IMC proteins, and several myosins (S4 Table). When these hits were evaluated based on normalized spectral abundances, the patterns for CaM1-BirA and CaM3-BirA were more similar to each other (e.g. 6 of the top 10 labeled proteins overlapped) (S4 Table). By contrast, the labeling pattern of CaM2-BirA was more diverse (S4 Table), although it included many of these same apical components at lower frequency, likely reflecting the fact that it is localized both at the apical end and in the cytosol (see Fig 1B). It is interesting to note that MyoH, and one of its light chains known as MLC7, were among the top 15 most abundant proteins labeled by CaM1-BirA and CaM3-BirA (S4 Table, Fig 7B). CaM1-BirA labeled MyoA at a higher level than the either CaM2-BirA or CaM3-BirA, suggesting it may also interact with MyoA, a myosin localized at the plasma membrane [70]. Although it is difficult to compare the frequencies of different hits directly due to differences in protein target abundance and efficiency of detection, these findings are consistent with CaM1 and CaM3 interacting with a number of apical complex proteins, including MyoH.

**Table 1:** Phenotypic comparison among *T*. *gondii* mutant lines.

	Parental line	cam2^KO^/CaM1-AID^1^	CaM3-AID^1^	MyoH knockdown^2^
Plaque formation	+	-	-/+	-
Conoid extrusion	+	+	+	+
Apical organelle distribution	+	+	+	+
Replication	+	+	+	+
Microneme secretion	+	+	+	+
Rhoptry secretion	+	+	+	+
Egress	+	-/+	-/+	-
Invasion	+	-	-/+	-
Motility	+	-	-/+	-
Invasion past the junction	+	-	-/+	-

+, phenotype normal; -, severe defect; -/+, moderate defect.

^1^ present study, phenotype refers to when grown in IAA

^2^ Reference 7

To examine the functional interaction between CaMs and MyoH, we generated a MyoH-AID line and separately tagged the CaM-like genes with 2Ty in this background using the CRISPR/Cas9 tagging technology. When MyoH was degraded by addition of auxin, CaM1-2Ty and CaM2-2Ty were mis-localized to the cytosol, and CaM3-2Ty was undetectable within 8 hr, as shown by IFA (Fig 7C and S4A Fig) and Western blotting (Fig 7D and S4B Fig). We also performed the reciprocal experiment and found that MyoH remained stable and apically localized when CaM3-AID or cam2^KO^/CaM1-AID were degraded by growth in auxin (S4C and S4D Fig). Another conoidal protein SAS6L was not affected by degradation of MyoH (Fig 7D). Collectively, these findings indicate that CaM1, CaM2 and CaM3 interact with the molecular motor MyoH at the conoid, likely regulating its activity in a calcium-dependent manner.

## Discussion

*Toxoplasma gondii* contains a large number of EF hand domain containing proteins including several calmodulin-like proteins that are concentrated at the conoid. Here we developed an efficient CRISPR-Cas9 mediated tagging technology, combined with a newly described system for auxin-induced degradation, to examine the localization and function of three CaM-like proteins localized to the conoid. The efficiency of these tools facilitated analysis of the functions of the essential *CaM3* gene as well as the synthetic lethal interaction between *CaM1* and *CaM2*. Phenotypic analyses revealed that all three CaMs participate in motility, egress, and invasion. CaM mutants were not defective in secretion from micronemes or rhoptries, but instead were stalled in entry into the host cells, a phenotype that mirrors that of the motor protein MyoH. Biochemical and cellular assays both indicate that these CaMs are in close proximity with MyoH at the conoid, where they may interact to coordinate early steps in motility. These findings expand the repertoire of essential proteins that are required for motility and cell invasion and highlight another layer of calcium regulation in this process through CaM-like proteins that may regulate myosin motors.

Our study on the molecular roles of *T*. *gondii* CaMs takes advantage of two recent developments for facilitating reverse genetics. First, the improved efficiency of CRISPR/Cas9 for targeted gene editing [31, 32] was adapted here to facilitate rapid tagging using a cassette approach where amplicons are generated from separate plasmids bearing distinct tags or markers using a common set of primers containing short homology regions for gene targeting. This approach eliminates the need for cloning separate plasmids for each gene of interest, and hence has advantages over previous methods, such as ligation-independent cloning [40]. We found that transfection of an amplicon bearing short homology regions (~40 bp) combined with a sgRNA CRISPR plasmid could be used to rapidly tag or disrupt genes of interest when applied in the ku80^KO^ background, which favors homologous recombination [40, 71]. The multiple tagging plasmids described here are easily adapted to other genes of interest by designing new primer pairs and modifying the CRISPR/Cas9 plasmid to contain a different sgRNA, as described previously [65]. Second, we adopted the plant-based auxin induced degradation system, which has significant advantages for analyzing biological functions as summarized below. First, the inducer, indoleacetic acid, is readily tolerated by *T*. *gondii* allowing high levels of compound to be used for either short term or longer culture periods without adverse effects, unlike Shield-1 that is typically used for FKBP-based protein degradation [35]. Second, induction of degradation is very rapid, within several hr for proteins studied here, although we have not made a direct comparison with the FKBP-based system for the same substrates used here. Using the auxin based system, the kinetics and extent of turnover differed between the reporter construct YFP-AID, which was fully degraded within 15 min, *vs*. the CaMS that required ≥ 3 hr for near complete loss of protein by Western blot. Whether this reflects intrinsic differences in the rate at which these protein substrates are recognized by the TIR1-SCF complex and hence targeted for degradation, or whether it is counterbalanced by difference in new protein synthesis is uncertain. Nonetheless for the substrates used here, the degradation was relatively rapid and allowed phenotypic evaluation within a few hr of addition of auxin. This rapid depletion at the protein level is much faster than gene deletion strategies such as inducible excision by Di-Cre [34] or Tet-off transcriptional control [10], which require decay of preexisting message, and protein turnover before phenotypes are evident. These combined features make the AID system ideal for studying essential genes (i.e. those refractory to gene deletion). We used this combined approach to reveal partially redundant functions for CaM1 and CaM2, either of which is dispensable but together their loss creates a lethal phenotype. Given the large number of genes that can be disrupted without apparent loss of function [33], such combined screening strategies are likely to be increasingly important for defining the function of genes that operate together.

Conformational changes that occur in conventional CaM when it binds to calcium affect its interaction with many binding partners, thereby modulating their functions [26]. In addition to a conventional and highly conserved CaM, *T*. *gondii* contains a number of proteins that contain four conserved EF hands in a conformation that is highly similar to CaM. An initial phylogenetic study described 13 CaM-like proteins in *T*. *gondii* and classified them as conventional CaM, centrin-like, or calmodulin like, based on similarity to animal, plant, and parasite proteins [27]. Conventional CaM, as well as CaM-like proteins containing a cluster of four EF hands, also function to control myosins, where they are typically referred to as myosin light chains (MLC) [72]. A further phylogenetic analysis of MLCs in *T*. *gondii* described an additional six MLCs that contained the typical assemblage of four EF hands, albeit most with degenerate calcium binding motifs, and unique N-terminal extensions [30]. The function of only a few of these CaM-like proteins has been addressed previously. Here we selected three CaMs that localize to the conoid and which have not been functionally analyzed before. These included MLC6 [30], which had previously been called CaM2 [16], and CaM1 [16], which is distinct from the conventional CaM protein described above, and a CaM-like protein (TGME49_026040) [30], referred to as CaM3 here. All three of these CaM-like proteins were found in the conoid-enriched fraction in a previous proteomics study [16], suggesting they may play important roles in this unique apical structure.

We utilized an efficient CRISPR/Cas9 mediated gene tagging approach combined with auxin-induced degradation to define the function(s) of CaM1, CaM2, and CaM3 in *T*. *gondii*. Degradation of CaM3-AID, or of CaM1-AID in a cam2^KO^ background led to a profound decrease in plaquing although this was not due to a block in replication. This growth defect was also not due to differences in the biosynthesis or secretion of micronemes or rhoptries, compartment that are needed for cell invasion. Instead, the CaM mutants showed defects in motility, impaired egress, and a strong block in cell invasion. Closer examination revealed that CaM degradation mutants were able to form a tight junction as revealed by RON4 staining, and secrete the contents of rhoptries to form evacuoles, but were unable to move past the junction and enter into the host cell. These phenotypes closely mirror those of MyoH, a motor protein implicated in translocation of micronemal adhesins from the apical tip, where they are first released, along the length of the conoid to the collar region where MyoA takes over this function [7]. Consistent with this prediction, all three CaMs largely colocalized with MyoH to the tip of the conoid, as shown by super-resolution microscopy. When used for permissive biotin proximity labeling, all three CaMs also labeled a large number of substrates, many of which are located at the apical complex, including MyoH and MLC7. Support for the hypothesis that CaMs may interact with MyoH was provided by the finding that CaM1 and CaM2 were mislocalized, and CaM3 was unstable, when MyoH was degraded using an AID-degradation domain fusion. However, in reciprocal experiments, the stability of MyoH was not affected by loss of CaM1, CaM2, or CaM3. This finding may be due to the insoluble nature of MyoH, which is believed to be tethered to the tubulin-rich conoid via its ATS domain [7].

Apicomplexans contain an expanded family of myosins, including type XIV myosins, which are uniquely shared by members of this phylum and ciliates [73]. *Toxoplasma gondii* contains 11 myosins, the functions of which have been defined for MyoA in gliding motility [10, 51], MyoB/C in cell division [74], MyoD that is localized to the plasma membrane but is nonessential *in vitro* [30], MyoF that mediates apicoplast inheritance [75], and MyoH that is involved in an early stage of cell invasion [7]. The expansion of myosins seen in *T*. *gondii* is accompanied by an expansion of CaM-like proteins [27], many of which appear to function as MLCs [30]. Several MLCs were previously localized to the plasma membrane (i.e. MLC1, MLC2) [5, 30], where they interact with and anchor MyoA and MyoD, respectively. Two different MLCs were shown to regulate the stability and activity MyoA [76], and based on these activities they were considered essential light chains (ELC). Interestingly, ELC1 and ELC2 appear to be functionally redundant as conditional shutdown (i.e. using the Tet-off system) of either gene alone did not reveal a phenotype, however conditional knockdown of both ELC1 and ELC2 together resulted in inhibition of invasion and egress ([76]. MLC1 also associates with MyoA anchoring it to the membrane [51] and regulating basal motor function [77]. In the present study our findings also suggest that that CaM1 may interact with MyoA, suggesting that these proteins together regulate this motor protein.

MyoH has eight IQ domains, a motif that is recognized by the EF hand domain in MLCs [72]. Three different MLCs were previously localized to the conoid (i.e. MLC3, MLC5 and MLC7) and two of these (i.e. MLC5, MLC7) were shown to interact with MyoH based on pulldowns [7]. Depletion of MyoH in a Tet-off system resulted in MLC5 losing its apical distribution and becoming cytosolic, while MLC7 retained its apical location, but decreased in expression level [7]. Importantly, when both MLC5 and MLC7 were disrupted using CRISPR/Cas9, there was no loss of parasite fitness, as revealed by plaque assay [7]. Pull-down studies also suggest that MLC1 also associates with MyoH, despite the fact that MLC1 is not apically concentrated [7].

In the present study, we utilized a combination of high-resolution microscopy and permissive biotin proximity labeling to demonstrate that CaM1, CaM2, and CaM3 colocalize and likely interact with MyoH. Our genetic disruption studies corroborate these findings and indicate that CaM3 is essential for the function of MyoH, while either CaM1 or CaM2 are required. However, these interactions are not exclusive, as CaMs labeled a wide range of other substrates when fused to the permissive biotin ligase BirA. Similarly, calmodulin is known to interact with a wide range of proteins, while also serving as a light chain for many non-conventional myosins in vertebrate cells [26]. As such, our findings are consistent with the hypothesis that CaM1, CaM2 and CaM3 in *T*. *gondii* interact with MyoH, possibly serving as regulatory light chains. Conserved calcium-binding residues in the EF hands of both CaM1 and CaM2 were required to rescue function, implying that their functions are calcium-dependent. In contrast, CaM3 contains entirely degenerate EF hands, and therefore is unlikely to bind calcium and may instead play a structural role. However, direct demonstration of interaction between the CaMs studied here and MyoH, including potential roles in regulating its activity or stability will require further study.

MyoH orthologues [73] and a number of more divergent MLCs [30] are found in the coccidian group of apicomplexans and these organisms also share the highly specialized conoid structure present in *T*. *gondii*. This conservation suggests that the functions defined here for *T*. *gondii* may extend to other coccidians. More distant MyoH orthologs also exist in piroplasms such as *Theileria* and *Babesia* [73], despite the fact that they do not contain the elaborate conoid structure seen in coccidians [15]. Hence MyoH orthologs may adopt different functions depending on the organism where they are found. Our findings add to existing studies on MyoH in *T*. *gondii* and suggest it may interact and be regulated by additional CaM-like proteins. These findings suggest that the expansion of CaM-like proteins in the *T*. *gondii* genome is an adaptation to regulate the many diverse myosins that they express. Moreover, they provide another layer for calcium-mediated control of motility by regulating motor proteins involved in adhesion translocation.

## Supporting information

S1 Text Extended methods. (DOCX)

S1 Table List of lines used. (DOCX)

S2 Table List of plasmids used. (DOCX)

S3 Table List of primers used. (DOCX)

S4 Table List of MS hits identified in biotinylation by BirA fusion proteins. (XLSX)

S1 Fig Diagram of CRISPR tagging technology. **A**. Schematic illustration of CRISPR tagging technology developed for *T*. *gondii*. Generic tagging plasmids that served as PCR templates for generation of gene-specific amplicons contained a linker (L, red box) and tags (green box) followed by a generic stop codon (gray box with s) and the *HXGPRT* 3’UTR (yellow box). A resistance marker expression cassette encoding HXGPRT, flanked with loxP sites, was included in the tagging plasmids, as illustrated. Amplicons were generated with a pair of primers incorporating the short homology HR1 (purple for the endogenous locus and red for the L region matching the Linker in the forward primer) and short homology HR2 (blue for the endogenous locus and black for the T region matching the T7 promoter (black) in the reverse primer) for a gene of interest. A Cas9-sgRNA 3’ plasmid that targeted close to the stop codon (gray box with s) of a specific gene of interest was combined with a gene-specific amplicon and co-transfected into a recipient line and transformants were selected with MPA and Xa as described in the methods. S in the gray boxes indicates a stop codon; U6p, RNA U6 promoter; scaffold, sgRNA scaffold; 3’UTR, *HXGPRT* 3’UTR. **B**. Example for the design of sgRNA 3’ and short homology region amplicons (HR1, HR2) for the gene encoding MyoH. The location in the sequence of the HR1 is shown in purple, the HR2 region in blue, and the sgRNA 3’ in orange. The Cas9 cleavage site is marked with a blue arrow. The stop codon is indicated by red lettering. **C**. Generation of a Cas9-sgRNA 3’ plasmid using Q5 DNA mutagenesis. A Cas9-sgRNA plasmid targeting the *UPRT* gene served as a DNA template for the Q5 mutagenesis reaction. The forward primer (sgRNA F) incorporated the MyoH sgRNA 3’ region and an adjacent region matching the sgRNA scaffold. The reverse primer (sgRNA R) was located just outside of the *UPRT* sgRNA on the reverse strand. **D**. Schematic of a variety of tagging plasmids used here. Different tags were integrated between the Linker and the *HXGPRT* selection cassette, providing various choices for generating tagging amplicons for a specific gene using the same pair of primers. The red boxes indicate the Linker, and the black box indicates a stop codon. **E.** Expanded diagram of the LoxP flanked *HXGPRT* cassette used in the tagging plasmids. The 5’ and 3’ regulatory regions from the *DHFR* gene were used to drive expression of HXGPRT.
(TIF)

S2 Fig Generation and verification of cam2^KO^*/*CaM1-AID, CaM3-AID, cam1^KO^/CaM2-AID transgenic parasites in the TIR1 parental line. **A and B.** Schematic of generation and diagnostic PCR for validating clones of cam2^KO^*/*CaM1-AID (A) and CaM3-AID lines (B). The cam2^KO^/CaM1-AID line (A) was generated first (step 1) by deletion of *CaM2* using the double sgRNA strategy in the TIR1 parental line followed by (step 2) CRISPR tagging at *CaM1* C-terminus with AID. The CaM3-AID line (B) was generated by CRISPR tagging at the C-terminus with AID. The resistance markers encoding DHFR or HXGPRT were excised by transfection of a Cre-GFP plasmid. Diagnostic PCR was performed using primers shown in the diagram that includes the PCR product sizes. WT, the TIR1 parental line; AID, cam2^KO^*/*CaM1-AID (A) and CaM3-AID (B); CDPK1 was used as PCR control. **C**. Generation and verification of the cam1^KO^*/*CaM2-AID line using a similar strategy to that described above, except cam1 was deleted and CAM2 was tagged with AID. Diagnostic PCR was performed using primers shown in the diagram that includes the PCR product sizes. WT, TIR1 parental line; AID, cam1^KO^/CAM2-AID. **D.** Degradation efficiency of AID-tagged CaM2 in the cam1^KO^/CaM2-AID line cultured for different periods of time in media with addition of auxin (500 μM) or 0.1% ethanol (vehicle). Samples were resolved with SDS-PAGE, blotted and detected with anti-HA (mouse) and anti-aldolase (rabbit), and probed with Licor IR-dye conjugated secondary antibodies. **E.** Plaque formation by the cam1^KO^*/*CAM2-AID line grown in D10 culture medium containing auxin (500 μM) (+IAA) or 0.1% ethanol (-IAA). Parasites were grown for 7 days, stained with Crystal violet, and plates scanned to generate the image. Scale bar = 0.5 cm.
(TIF)

S3 Fig Biotinylation of proximal proteins using BirA-fusions of CaM1, CaM2 and CaM3. **A.** Fusion of BirA tag with endogenous protein labeling of interacting or proximal proteins in the presence of exogenous D-biotin in media. Biotinylated proteins were affinity purified using streptavidin conjugated magnetic beads from SDS-denatured lysis, and identified by mass spectrometry. **B.** Western blot confirmation of BirA fusion lines of CaM1-BirA-3xHA, CaM2-BirA-3xHA, and CaM3-BirA-3xHA. Western blot was detected with antibodies mouse-anti-HA (HA) and rabbit anti-aldolase (ALD), followed with Licor IR-dye conjugated secondary antibodies. **C.** Immunofluorescence confirmation of biotinylation in CaM1-BirA, CaM2-BirA and CaM3-BirA lines. Parasites were grown in media containing D-Biotin for 24 hr, fixed, permeabilized, and stained with antibodies rabbit anti-HA, followed with anti-rabbit Alexa Fluor-594 and streptavidin-Alexa Fluor-488. The parental line ku80^KO^ served as a control. Endogenous biotin containing proteins were detected in all lines, while apical CaM-dependent labeling was only seen in the BirA fusion lines. Scale bar = 2 μm.
(TIF)

S4 Fig Association of CaM1, CaM2. and CaM3 with MyoH at the conoid. There was a loss of conoid localization or disappearance of CaMs upon depletion of MyoH (A, B). In contrast, MyoH was stable in the CaM2^KO^CaM1-AID and CaM3-AID strains following auxin degradation (C, D). **A**. CaM1, CaM2 and CaM3 were tagged with 2xTy in the MyoH-AID line. Parasites were grown on coverslips with HFF for 8 hr with 500 μM IAA (+IAA) or ethanol alone (-IAA), fixed, and stained by IFA using mouse anti-Ty antibodies and rabbit anti-aldolase antibodies followed by anti-mouse Alexa Fluor-488 and anti-rabbit Alexa Fluor-594. The protein SAS6L was used as control. Scale bar = 2μm. **B.** Western blot detection of CaMs tagged with Ty in the MyoH-AID line. Parasites were grown for different treatment times with IAA (500 μM) or 0.1% ethanol (vehicle). Parasites were resolved with SDS-PAGE, blotted, and probed with mouse anti-HA, mouse anti-Ty, and rabbit anti aldolase, followed by Licor IR-dye conjugated secondary antibodies. **C**. MyoH was tagged with 2Ty in the AID strains. Parasites were treated with either IAA (500 μM) or 0.1% ethanol (vehicle) for 2 days and Western blotted with anti-HA (to detect the AID fusion), anti-Ty (to detect MyoH), and anti-aldolase (ALD, control) antibodies followed by Licor IR-dye conjugated secondary antibodies. **D**. Immunofluorescence microscopy was performed with parasites grown for 24 hr with either IAA (500 μM) or 0.1% ethanol (vehicle) and stained using anti-Ty (stained green) and anti-GAP45 (stained red) antibodies. Scale bar = 2μm.
(TIF)
