# Supplementary material for: Calmodulin-like proteins localized to the conoid regulate motility and cell invasion by Toxoplasma gondii
Source: PLoS Pathog. 2017 May 5;13(5):e1006379. doi: 10.1371/journal.ppat.1006379 (PMC5435356; doi:10.1371/journal.ppat.1006379)
Supplement: S1 Text — (DOCX) [file ppat.1006379.s001.docx]

**Material and Methods**

**Plasmids used for epitope tagging**

A series of plasmids for epitope tagging of genes of interest was generated using the following strategy: A *HXGPRT* expression cassette flanked with LoxP sites was amplified with primers listed in Set 1 (Table S3) and cloned into the previously described pLic-3xHA-HXGPRT plasmid [1] using restriction sites *HindIII* and *NotI* to generate the plasmid pLic-3xHA-HXGPRT-LoxP. A DNA amplicon containing the Linker-3xHA-HXGPRT 3’UTR was generated from a 3xHA-containing plasmid pMAPK-HA [2] using primers Set 2 (Table S3), and cloned into pLic-3xHA-HXGPRT-LoxP using restrictions sites *NheI* and *XhoI*, producing the plasmid named pLinker-3xHA-HXGPRT-LoxP (Table S2). The pLinker-3xHA-HXGPRT-LoxP was modified to generate pLinker-6xHA-HXGPRT-LoxP by removing the stop codon for 3xHA and by introducing a restriction site *AscI* into pLinker-3xHA-HXGPRT-LoxP using a Q5 mutagenesis kit with primer Set 3, and by following with a cloning of 3xHA-HXGRPT 3’UTR from pLic-BirA-3xHA-DHFR [3] into the mutated pLinker-3xHA-HXGPRT-LoxP using primer Set 4 and restriction sites *AscI* and *XhoI* (Table S2,3). The plasmid pLinker-6xHA-HXGPRT-LoxP was used as a DNA template to generate another two plasmids called pLinker-2xTy-HXGPRT-LoxP and pLinker-myc-HXGPRT-LoxP, using a Q5 DNA mutagenesis kit (New England Biolabs) with primers Set 5 and Set 6, respectively (Table S3). The plasmid pLinker-BirA-3xHA-HXGPRT-LoxP was generated by cloning a DNA fragment containing Linker-BirA-3xHA-HXGPRT 3’UTR amplified from the plasmid pLic-BirA-3xHA-DHFR [3] into pLinker-6xHA-HXGPRT-LoxP with primers Set 7 (Table S3) using restriction sites *NheI* and *XhoI*. The plasmid pLinker-AID-3xHA-HXGPRT-LoxP was generated by cloning a Linker-AID DNA fragment amplified from the plasmid pG362P [4] with primers Set 8 (Table S3) into pLinker-6xHA-HXGPRT-LoxP using *NheI* and *AscI* restriction sites. The 3xHA tag in pLinker-AID-3xHA-HXGPRT-LoxP was removed and replaced with Ty using a Q5 mutagenesis kit with primer Set 9 (Table S3), creating another AID plasmid pLinker-AID-Ty-HXGPRT-LoxP (Table S2). The Linker-X-HXGPRT 3’UTR segment was sequenced to confirm the correct coding frame in these plasmids. These tagging plasmids pLinker-X-HXGPRT-LoxP are listed in Table S2, and they all have a common sequence - Linker (gctagcAAGGGCTCGGGCTCGACCCAGCTG, encoding ASKGSGSTQL), which served as an upstream PCR matching site named L (gctagcAAGGGCTCGG) to ensure that CRISPR tagging amplicons were in frame with targeting genes and the appropriate epitope tag in the plasmid. The downstream PCR matching site named T (AATACGACTCACTATAGG) was located at the reverse DNA strand of T7 promoter after the resistance marker HXGPRT. In some experiments, the HXGPRT-LoxP cassette was replaced with a cassette containing LoxP-DHFR-LoxP excised from pDHFR-LoxP (see below) using restriction sites *HindIII* and *NotI*.

**Endogenous genetic tagging of *T. gondii* using CRISPR technology**

A downstream sgRNA (named sgRNA 3’) designed to target close to the stop codon (i.e. within 200 bp) (Fig. S1A and S1B) was used to create a double strand break for endogenous tagging using short homologous regions flanking a tagging cassette. CRISPR-Cas9 sgRNA 3’ plasmids (Table S2) specifically targeting genes of interest were generated by a Q5 DNA mutagenesis kit using primers listed in Table S3 (Fig. S1C). To generate amplicons flanked with short homology regions, the forward primer HR1-L was designed to incorporate 40-42 bp of short homology region (HR1) matching upstream of the stop codon of a specific gene of interest and a 17-bp sequence corresponding to the L region of the tagging plasmid. Similarly, the reverse primer HR2-T was designed to incorporate 40-42 bp of short homology region (HR2) downstream of the sgRNA 3’cleavage site on the reverse DNA strand and containing the T region of the targeting plasmid. The details of the HR1, HR2, and sgRNA 3’ designing were illustrated in Fig. S1 using MyoH as an example. DNA amplicons generated with each pair of primers HR1-L and HR2-T from pLinker-(epitope tag of interest)-HXGPRT-LoxP tagging plasmids were combined with the corresponding CRISPR-Cas9-sgRNA 3’ targeting plasmids, and co-transfected into a recipient line. Primers HR1-L and HR2-T for tagging each of the genes studied here with a variety of different tags are listed in Table S3.

**Generation of knockouts using a double CRISPR sgRNA strategy**

Knockouts were generated using a double sgRNA strategy for CRISPR/Cas9 as described previously [5]. In brief, a double CRISPR/Cas9 sgRNA plasmid for each gene of interest was combined with an amplicon containing a LoxP-DHFR-mCherry-LoxP cassette flanked by short homology regions (HRs) and co-transfected into the ku80^KO^ line followed by Pyr selection. A CRISPR/Cas9 sgRNA 5’ was designed targeting close to the start codon of a gene of interest, and the targeting plasmids pCas9 sgRNA 5’ (Table S2) were generated using primers listed in Table S3. The U6:sgRNA 5’ cassettes were amplified using primer Set 10 in Table S3 and cloned into their corresponding plasmids CRISPR-Cas9 sgRNA 3’ (Table S2) using restriction sites *XhoI* and *KpnI*. Amplicons with short homologies flanking with the LoxP-DHFR-mCherry-LoxP cassette were generated with primers M and HR2-T (Table S3) for each gene.

**Transfection and subcloning of transgenic *T. gondii* lines**

For epitope tagging, amplicons generated with primers HR1-L and HR2-T contain a Linker-(epitope tag of interest)-HXGPRT-LoxP cassette flanked with short homology regions (HR1 and HR2). Amplicons were purified and combined with their corresponding pCas9-sgRNA 3’ plasmids. Approximately 2 μg of amplicon and 10-20 µg of a corresponding pCas9-sgRNA 3’ targeting plasmid were combined with 200 µl containing 3-5 x 10^6^ recipient parasites in cytomix buffer, as described previously [5]. Following electroporation, cells were inoculated to HFF monolayers grown in T-25 flasks, and cultured for 24 hr at 37°C with 5% CO_2_, before addition of mycophenolic acid (MPA) and 6-xanthine (6-Xa) (25 µg/ml and 50 µg/ml, respectively). Selection pools appeared in 1-2 weeks and parasites were subcloned on HFF monolayers grown in 96-wells plates cultured as above. For test of gene essentiality, amplicons containing a LoxP-DHFR-mCherry-LoxP cassette generated with primers M and HR2-T from pDHFR-mCherry-LoxP [6] and flanked by short homology regions were combined with their corresponding CRISPR double sgRNA 5’/3’ targeting plasmids and the selection was performed with 3 µM Pyr. To generate lines expressing the TIR1 protein we used the plasmid pTUB1-OsTIR1-3Flag-CAT (Table S2). To the TIR1 parental line, we added a plasmid to express the AID protein using the plasmid pTUB1-YFP-AID-3HA-HXGPRT-LoxP (Table S2). To generate complements in the cam2^KO^*/*CaM1-AID line, we expressed CaM1-Ty or CaM2-Ty or various mutant forms using the plasmids defined in Table S2. Approximately 20 µg of purified plasmids were electroporated into recipient lines and parasites were selected with chlorophenical, MPA/6-Xa and Pyr, respectively.

**Generation of double knockouts**

Single gene knockouts *∆cam1* or *∆cam2* that were generated with a previously developed CRISPR double sgRNA strategy contained the DHFR Pyr-resistance cassette. The DHFR marker was removed by electroporation of 50 µg of pmin-Cre-GFP [7] and parasites were cloned on day 2 or 3 without drug selection. Clones were screened by PCR using primers p1 and p2 (Table S3). Sensitivity to 3 μM Pyr was confirmed, before clones were used for a second round of transfection to generate double knockouts using CRSIRP/Cas9 targeting plasmids described above.

**Single clone screening and diagnostic PCR**

Cell lysates of parasite clones were screened using primers designed to detect transgenic parasites with the correct genotypes. In each case, primer p1 is a forward primer ~1200bp upstream of the CRISPR sgRNA 5’ cleavage site. Primer p2 is a reverse primer ~100-500bp downstream of the CRISPR Cas9-sgRNA 3’ targeting site that was also used for tagging. Primer p3 is a forward primer ~ 500 bp upstream of the stop codon. Primer p corresponds to a common sequence in the *HXGPRT* 3’UTR. To screen clones for the mutants ∆*cam1*, ∆*cam2*, and ∆c*am3*, primers p1/p2 were used to test the insertion of the *DHFR* cassette in the respective loci, while primers p2/p3 were used to test the absence/presence of the endogenous genes. For screening the line cam2^KO^/CaM1-AID, the *∆cam2* deletion was first tested in the same way, then primers p2/p3 were used to test the insertion of Linker-AID-HXGPRT-LoxP, and primers p3/p were to test the integration of the AID fusion. For the CaM3-AID line, similar PCR reactions were performed to confirm AID tagging. Similar procedures were applied to screen clones of cam1^KO^/CaM2-AID-3xHA, and the MyoH-AID. The primers for diagnostic PCR are listed in Table S3.

**Plasmid generation for complementation lines**

The plasmid pDHFR-LoxP (Table S2) was generated by a Q5 DNA mutagenesis kit using primer set 11 (Table S3, to remove mCherry encoding sequence from pDHFR-mCherry-LoxP [6]. A DNA cassette containing multiple restrictions sites including *AvrI*, *NheI*, *ApaI*, and a DHFR 3’UTR was amplified from pDHFR-LoxP using a primer Set 12 (Table S3, and cloned into the vector pDHFR-LoxP with restrictions sites *NotI* and *SpeI*, which were created by a Q5 mutagenesis using primer set 13 at the pDHFR-LoxP, creating pDHFR-LoxP-MCS (Table S2). A DNA fragment from pLinker-2xTy-HXGPRT-LoxP was excised using the restriction enzymes *ApaI* and *XhoI* and cloned into pDHFR-LoxP-MCS, creating pDHFR-LoxP-2xTy (Table S2), which was used to generate complementation plasmids. For CaM1, primer set 14 was designed to amplify the 5’UTR and the CDS in front of the stop codon and this fragment was cloned into pDHFR-LoxP-2xTy between *NotI* and *ApaI*, creating a plasmid pDHFR-LoxP-CaM1-2xTy (Table S2). Mutations in the calcium binding aspartic residues in EF hands were generated using primer set 15 and set 16 (Table S3) by Q5 mutagenesis, generating plasmids pDHFR-LoxP-CaM1-2xTy EF1m and pDHFR-LoxP-CaM1-2xTy EF2m (Table S2). The plasmid pDHFR-LoxP-CaM1-2xTy EF2m was used to generate mutations in the EF hand 1 using primer set 15, producing the plasmid pDHFR-LoxP-CaM1-2xTy-EF1/2m (Table S2). For CaM2, the 5’UTR from CaM1 was first cloned into pDHFR-LoxP-2xTy with primer set 17 (Table S3) using restriction sites *NotI* and *AvrI*. The CaM2 CDS was then amplified with primer set 18 (Table S3) and cloned into *AvrI* and *ApaI*, creating plasmid pDHFR-LoxP-CaM2-2xTy (Table S2). The plasmid pDHFR-LoxP-CaM2-2xTy was used to generate mutations at aspartic residues using primer set 19 and set 20 (Table S3) using the Q5 mutagenesis kit, creating plasmids pDHFR-LoxP-CaM2-2xTy EF1m and pDHFR-LoxP-CaM2-2xTy EF2m (Table S2). All plasmids were sequenced using a primer CaM Seq (Table S3), to verify the sequences and mutations by Sanger sequencing.

**References**

1. Huynh MH, Carruthers VB. Tagging of endogenous genes in a *Toxoplasma gondii* strain lacking Ku80. Eukaryot Cell. 2009;8(4):530-9.

2. Brown KM, Suvorova E, Farrell A, McLain A, Dittmar A, Wiley GB, et al. Forward genetic screening identifies a small molecule that blocks Toxoplasma gondii growth by inhibiting both host- and parasite-encoded kinases. PLoS Pathog. 2014;10(6):e1004180.

3. Chen AL, Kim EW, Toh JY, Vashisht AA, Rashoff AQ, Van C, et al. Novel components of the Toxoplasma inner membrane complex revealed by BioID. MBio. 2015;6(1):e02357-14.

4. Philip N, Waters AP. Conditional Degradation of Plasmodium Calcineurin Reveals Functions in Parasite Colonization of both Host and Vector. Cell Host Microbe. 2015

5. Long S, Wang Q, Sibley LD. Analysis of Noncanonical Calcium-Dependent Protein Kinases in Toxoplasma gondii by Targeted Gene Deletion Using CRISPR/Cas9. Infect Immun. 2016;84(5):1262-73.

6. Sullivan WJ, Jr., Smith CK, 2nd. Cloning and characterization of a novel histone acetyltransferase homologue from the protozoan parasite *Toxoplasma gondii* reveals a distinct GCN5 family member. Gene. 2000;242(1-2):193-200.

7. Heaslip AT, Nishi M, Stein B, Hu K. The motility of a human parasite, *Toxoplasma gondii*, is regulated by a novel lysine methyltransferase. PLoS Pathog. 2011;7(9):e1002201.

8. Bastin P, Bagherzadeh Z, Matthews KR, Gull K. A novel epitope tag system to study protein targeting and organelle biogenesis in *Trypanosoma brucei*. Molec Biochem Parasitol. 1996;77:235-9.

9. Roux KJ, Kim DI, Raida M, Burke B. A promiscuous biotin ligase fusion protein identifies proximal and interacting proteins in mammalian cells. J Cell Biol. 2012;196(6):801-10.

10. Paul AS, Saha S, Engelberg K, Jiang RH, Coleman BI, Kosber AL, et al. Parasite Calcineurin Regulates Host Cell Recognition and Attachment by Apicomplexans. Cell Host Microbe. 2015;18(1):49-60.

11. Nebl T, Prieto JH, Kapp E, Smith BJ, Williams MJ, Yates JR, 3rd, et al. Quantitative in vivo analyses reveal calcium-dependent phosphorylation sites and identifies a novel component of the Toxoplasma invasion motor complex. PLoS Pathog. 2011;7(9):e1002222.

12. Williams MJ, Alonso H, Enciso M, Egarter S, Sheiner L, Meissner M, et al. Two Essential Light Chains Regulate the MyoA Lever Arm To Promote Toxoplasma Gliding Motility. MBio. 2015;6(5):e00845-15.

13. Hu K, Johnson J, Florens L, Franholz M, Suravajjala S, Dilullo C, et al. Cytoskeletal components of an invasion machine - the apical complex of *Toxoplasma gondii*. PLoS Pathogens. 2006;2:121-38.

14. Frenal K, Polonais V, Marq JB, Stratmann R, Limenitakis J, Soldati-Favre D. Functional dissection of the apicomplexan glideosome molecular architecture. Cell Host Microbe. 2010;8(4):343-57.

15. Polonais V, Javier Foth B, Chinthalapudi K, Marq JB, Manstein DJ, Soldati-Favre D, et al. Unusual anchor of a motor complex (MyoD-MLC2) to the plasma membrane of Toxoplasma gondii. Traffic. 2011;12(3):287-300.

16. Graindorge A, Frenal K, Jacot D, Salamun J, Marq JB, Soldati-Favre D. The Conoid Associated Motor MyoH Is Indispensable for Toxoplasma gondii Entry and Exit from Host Cells. PLoS Pathog. 2016;12(1):e1005388.

17. Hu K. Organizational changes of the daughter basal complex during the parasite replication of Toxoplasma gondii. PLoS Pathog. 2008;4(1):e10.

18. Leport C, Franck J, Chene G, Derouin F, Ecobichon JL, Pueyo S, et al. Immunoblot profile as predictor of toxoplasmic encephalitis in patients infected with human immunodeficiency virus. Clinical and Diagnostic Laborstory Immunology. 2001;8:579-84.
