# Supplementary material for: Calmodulin-like proteins localized to the conoid regulate motility and cell invasion by Toxoplasma gondii
Source: PLoS Pathog. 2017 May 5;13(5):e1006379. doi: 10.1371/journal.ppat.1006379 (PMC5435356; doi:10.1371/journal.ppat.1006379)
Supplement: S1 Table — (DOCX) [file ppat.1006379.s002.docx]

**S1 Table**. List of lines used.

|  | Line | Genotype | Resistance marker | Associated plasmid |
| --- | --- | --- | --- | --- |
| 1 | RHku80^KO^ | RH*∆ku80∆hxgprt* | NO |  |
| 2 | CaM1-6HA | RH*∆ku80∆hxgprt; CaM1-6HA, DHFR-TS:HXGPRT* | HXGPRT | p1, p13 |
| 3 | CaM2-6HA | RH*∆ku80∆hxgprt; CaM2:6HA, DHFR-TS:HXGPRT* | HXGPRT | p4, p13 |
| 4 | CaM3-6HA | RH*∆ku80∆hxgprt; CaM3-6HA, DHFR-TS:HXGPRT* | HXGPRT | p7, p13 |
| 5 | cam1^KO^ | RH*∆ku80∆hxgprt; ∆cam1::DHFR-TS:DHFR-mCherry* | DHFR | p3 |
| 6 | cam2^KO^ | RH*∆ku80∆hxgprt; ∆cam2::DHFR-TS:DHFR-mCherry* | DHFR | p6 |
| 7 | TIR1 | RH*∆ku80∆hxgprt; TUB1:OsTIR1-3FLAG,SAG1:CAT* | CAT | p18 |
| 8 | YFP-AID-3HA | RH*∆ku80∆hxgprt; TUB1:OsTIR1-3FLAG, SAG1:CAT/TUB1:YFP-AID-3HA,DHFR-TS:HXGPRT* | CAT, HXGPRT | p18, p19 |
| 9 | cam1^KO^/TIR1 | RH*∆ku80∆hxgprt; TUB1:OsTIR1-3FLAG, SAG1:CAT; ∆cam1::DHFR-TS:DHFR-mCherry* | CAT, DHFR | p18, p3 |
| 10 | cam2^KO^/TIR1 | RH*∆ku80∆hxgprt; TUB1:OsTIR1-3FLAG, SAG1:CAT; ∆cam2::DHFR-TS:DHFR-mCherry* | CAT,  DHFR | p18, p6 |
| 11 | cam2^KO^/CaM1-AID | RH*∆ku80∆hxgprt; TUB1:OsTIR1-3FLAG, SAG1:CAT; ∆cam2; CaM1-AID-3HA* | CAT | p18, p6, p1, p14 |
| 12 | cam1^KO^/CaM2-AID | RH*∆ku80∆hxgprt; TUB1:OsTIR1-3FLAG, SAG1:CAT; ∆cam1::DHFR-TS:DHFR-mCherry; CaM2-AID-3HA, DHFR-TS:HXGPRT* | CAT ,  HXGPRT,  DHFR | p18, p3, p6, p14 |
| 13 | CaM3-AID | RH*∆ku80∆hxgprt; TUB1:OsTIR1-3FLAG, SAG1:CAT; CaM3-AID-3HA* | CAT | p18, p7, p14 |
| 14 | cam2^KO^/CaM1-AID /CaM1-Ty | RH*∆ku80∆hxgprt; TUB1:OsTIR1-3FLAG, SAG1:CAT; ∆cam2; CaM1-AID-3HA/CaM1:CaM1-2Ty, DHFR-TS:DHFR* | CAT, DHFR | p18, p6, p1, p14, p24 |
| 15 | cam2^KO^/CaM1-AID /CaM1-Ty EF1m | RH*∆ku80∆hxgprt; TUB1:OsTIR1-3FLAG, SAG1:CAT; ∆cam2; CaM1-AID-3HA/CaM1:cam1^D38A,D40A,D42A^-2Ty, DHFR-TS:DHFR* | CAT, DHFR | p18, p6, p1, p14, p25 |
| 16 | cam2^KO^/CaM1-AID /CaM1-Ty EF2m | RH*∆ku80∆hxgprt; TUB1:OsTIR1-3FLAG, SAG1:CAT; ∆cam2; CaM1-AID-3HA/CaM1:cam1^D120A,D122A,D124A^-2Ty, DHFR-TS:DHFR* | CAT, DHFR | p18, p6, p1, p14, p26 |
| 17 | cam2^KO^/CaM1-AID /CaM1-Ty EF1/2m | RH*∆ku80∆hxgprt; TUB1:OsTIR1-3FLAG, SAG1:CAT; ∆cam2; CaM1-AID-3HA/CaM1:cam1^D38A,D40A,D42A, D120A,D122A,D124A^-2Ty, DHFR-TS:DHFR* | CAT, DHFR | p18, p6, p1, p14, p27 |
| 18 | cam2^KO^/CaM1-AID /CaM2-Ty | RH*∆ku80∆hxgprt; TUB1:OsTIR1-3FLAG, SAG1:CAT; ∆cam2; CaM1-AID-3HA/CaM1:CaM2-2Ty, DHFR-TS:DHFR* | CAT, DHFR | p18, p6, p1, p14, p28 |
| 19 | cam2^KO^/CaM1-AID /CaM2-Ty EF1m | RH*∆ku80∆hxgprt; TUB1:OsTIR1-3FLAG, SAG1:CAT; ∆cam2; CaM1-AID-3HA/CaM1:cam2^D13A,D15A,D17A^-2Ty, DHFR-TS:DHFR* | Cm, DHFR | p18, p6, p1, p14, p29 |
| 20 | cam2^KO^/CaM1-AID /CaM2-Ty EF2m | RH*∆ku80∆hxgprt; TUB1:OsTIR1-3FLAG, SAG1:CAT; ∆cam2; CaM1-AID-3HA/CaM1:cam2^D83A,D91A^-2Ty, DHFR-TS:DHFR* | CAT, DHFR | p18, p6, p1, p14, p30 |
| 21 | cam2^KO^/CaM1-AID /MIC2-GLuc-myc | RH*∆ku80∆hxgprt; TUB1:OsTIR1-3FLAG, SAG1:CAT; ∆cam2; CaM1-AID-3HA/TUB1:MIC2-GLuc-myc, DHFR-TS:HXGPRT* | CAT, HXGPRT | p18, p6, p1, p14, p20 |
| 22 | MIC2-Gluc-myc/TIR1 | RH*∆ku80∆hxgprt; TUB1:OsTIR1-3FLAG, SAG1:CAT/TUB1:MIC2-GLuc-myc, DHFR-TS:HXGPRT* | CAT, HXGPRT | p18, p20 |
| 23 | CaM3-AID/MIC2-Gluc-myc | RH*∆ku80∆hxgprt; TUB1:OsTIR1-3FLAG, SAG1:CAT; CaM3-AID-3HA/TUB1:MIC2-GLuc-myc, DHFR-TS:HXGPRT* | CAT, HXGPRT | p18, p7, p14, p20 |
| 24 | MyoH-AID | RH*∆ku80∆hxgprt; TUB1:OsTIR1-3FLAG, SAG1:CAT; MyoH-AID-3HA, DHFR-TS:HXGPRT* | CAT, HXGPRT | p18, p10, p14 |
| 25 | MyoH-AID/CaM1-Ty | RH*∆ku80∆hxgprt; TUB1:OsTIR1-3FLAG, SAG1:CAT; MyoH-AID-3HA, DHFR-TS:HXGPRT; CaM1-2Ty, DHFR-TS:DHFR* | CAT, HXGPRT, DHFR | p18, p10, p14, p1, p16 |
| 26 | MyoH-AID/CaM2-Ty | RH*∆ku80∆hxgprt; TUB1:OsTIR1-3FLAG, SAG1:CAT; MyoH-AID-3HA, DHFR-TS:HXGPRT; CaM2-2Ty, DHFR-TS:DHFR* | CAT, HXGPRT, DHFR | p18, p10, p14, p4, p16 |
| 27 | MyoH-AID/CaM3-Ty | RH*∆ku80∆hxgprt; TUB1:OsTIR1-3FLAG, SAG1:CAT; MyoH-AID-3HA, DHFR-TS:HXGPRT; CaM3-2Ty, DHFR-TS:DHFR* | CAT, HXGPRT, DHFR | p18, p10, p14, p7, p16 |
| 28 | MyoH-AID/SAS6L-Ty | RH*∆ku80∆hxgprt; TUB1:OsTIR1-3FLAG, SAG1:CAT; MyoH-AID-3HA, DHFR-TS:HXGPRT; SAS6L-2Ty, DHFR-TS:DHFR* | CAT, HXGPRT, DHFR | p18, p10, p14, p11, p16 |
| 29 | CaM1-BirA | RH*∆ku80∆hxgprt*; *CaM1-BirA-3HA, DHFR-TS:HXGPRT* | HXGPRT | p1, p17 |
| 30 | CaM2-BirA | RH*∆ku80∆hxgprt*; *CaM2-BirA-3HA, DHFR-TS:HXGPRT* | HXGPRT | P4, p17 |
| 31 | CaM3-BirA | RH*∆ku80∆hxgprt*; *CaM3-BirA-3HA, DHFR-TS:HXGPRT* | HXGPRT | P7, p17 |
| 32 | MyoH-3HA/CaM1-2Ty | RH*∆ku80∆hxgprt*; *MyoH-3HA, DHFR-TS:HXGPRT; CaM1-2Ty, DHFR-TS:DHFR* | DHFR,  HXGPRT | p18, p10, p12, p1, p16 |
| 33 | MyoH-3HA/CaM2-2Ty | RH*∆ku80∆hxgprt*; *MyoH-3HA, DHFR-TS:HXGPRT; CaM2-2Ty, DHFR-TS:DHFR* | DHFR,  HXGPRT | p18, p10, p12, p4, p16 |
| 34 | MyoH-3HA/CaM3-2Ty | RH*∆ku80∆hxgprt*; *MyoH-3HA, DHFR-TS:HXGPRT; CaM3-2Ty, DHFR-TS:DHFR* | DHFR,  HXGPRT | p18, p10, p12, p7, p16 |
