# Supplementary material for: Calmodulin-like proteins localized to the conoid regulate motility and cell invasion by Toxoplasma gondii
Source: PLoS Pathog. 2017 May 5;13(5):e1006379. doi: 10.1371/journal.ppat.1006379 (PMC5435356; doi:10.1371/journal.ppat.1006379)
Supplement: S2 Table — (DOCX) [file ppat.1006379.s003.docx]

**S2 Table**. List of plasmids used.

|  | Plasmid name | Genotype | Application (addgene) |
| --- | --- | --- | --- |
| p1 | pCas9-CaM1 sgRNA 3’ | *SAG1:Cas9-GFP^[18]^, U6:CaM1sgRNA 3’* | Tagging and deletion |
| p2 | pCas9-CaM1 sgRNA 5’ | *SAG1:Cas9-GFP, U6:CaM1sgRNA 5’* | Gene knockout |
| p3 | pCas9-CaM1 sgRNA 5’/3’ | *SAG1:Cas9-GFP, U6:CaM1sgRNA 5’, U6: CaM1sgRNA 3’* | Gene knockout |
| p4 | pCas9-CaM2 sgRNA 3’ | *SAG1:Cas9-GFP, U6:CaM2sgRNA 3’* | Tagging and deletion |
| p5 | pCas9-CaM2 sgRNA 5’ | *SAG1:Cas9-GFP, U6:CaM2sgRNA 5’* | Gene knockout |
| p6 | pCas9-CaM2 sgRNA 5’/3’ | *SAG1:Cas9-GFP, U6:CaM2sgRNA 5’, U6:CaM2sgRNA 3’* | Gene knockout |
| p7 | pCas9-CaM3 sgRNA 3’ | *SAG1:Cas9-GFP, U6:CaM3sgRNA 3’* | Tagging and deletion |
| p8 | pCaS9-CaM3 sgRNA 5’ | *SAG1:Cas9-GFP, U6:CaM3sgRNA 5’* | Gene knockout |
| p9 | pCaS9-CaM3 sgRNA 5’/3’ | *SAG1:Cas9-GFP, U6:CaM3sgRNA 5’, U6: CaM3sgRNA 3’* | Gene knockout |
| p10 | pCas9-MyoH sgRNA 3’ | *SAG1:Cas9-GFP, U6:MyoHsgRNA 3’* | Tagging |
| p11 | pCas9-SAS6LsgRNA 3’ | *SAG1:Cas9-GFP, U6:SAS6LsgRNA 3’* | Tagging |
| p12 | pLinker-3HA-HXGPRT-LoxP | *Linker-3HA, LoxP-DHFR-TS:HXGPRT-LoxP* | PCR template for 3HA tagging amplicons |
| p13 | pLinker-6HA-HXGPRT-LoxP | *Linker-6HA, LoxP-DHFR-TS:HXGPRT-LoxP* | PCR template for 6HA tagging amplicons **(86552)**86552 86552 |
| p14 | pLinker-AID-3HA-HXGPRT-LoxP | *Linker-AID-3HA, LoxP-DHFR-TS:HXGPRT-LoxP* | PCR template for AID-3HA tagging amplicons **(86553)** |
| p15 | pLinker-2Ty-HXGPRT-LoxP | *Linker-2Ty, LoxP-DHFR-TS:HXGPRT-LoxP* | PCR template for 2Ty tagging amplicons **(86664)** |
| p16 | pLinker-2Ty-DHFR-LoxP | *Linker-2Ty, LoxP-DHFR-TS:DHFR-LoxP* | PCR template for 2Ty tagging amplicons **(86670)** |
| p17 | pLinker-BirA-3HA-HXGPRT-LoxP | *Linker-BirA-3HA, LoxP-DHFR-TS:HXGPRT-LoxP* | PCR template for BirA-3HA tagging amplicon **(86668)** |
| p18 | pTUB1-OsTIR1-3FLAG-CAT | *TUB1:OsTIR1-3FLAG, SAG1:CAT* | Generation of TIR1 parental line |
| p19 | pTUB1-YFP-AID-3HA-HXGPRT-LoxP | *TUB1:YFP-Linker-AID-3HA, DHFR-TS:HXGPRT-LoxP* | Testing the efficiency of TIR1-AID system |
| p20 | pMIC2-GLuc-myc | *TUB1:MIC2-GLuc-myc, DHFR-TS:HXGPRT* | Microneme secretion |
| p21 | pDHFR-LoxP | *LoxP-DHFR-TS:DHFR-LoxP* | Cloning of CaM1 and CaM2 for complementation |
| p22 | pDHFR-LoxP-MSC | *LoxP-DHFR-TS:DHFR-LoxP, DHFR-TS:MSC* | Cloning of CaM1 and CaM2 for complementation |
| p23 | pDHFR-LoxP-2Ty | *LoxP-DHFR-TS:DHFR-LoxP, DHFR-TS:2xTy* | Cloning of CaM1 and CaM2 for complementation |
| p24 | pDHFR-LoxP-CaM1-2Ty | *LoxP-DHFR-TS:DHFR-LoxP, CaM1:CaM1-2Ty* | Complementation |
| p25 | pDHFR-LoxP-CaM1-2xTy EF1m | *LoxP-DHFR-TS:DHFR-LoxP, CaM1:CaM1^D38A,D40A,D42A^-2Ty* | Complementation |
| p26 | pDHFR-LoxP-CaM1-2xTy EF2m | *LoxP-DHFR-TS:DHFR-LoxP, CaM1:CaM1^D120A,D122A,D124A^-2Ty* | Complementation |
| p27 | pDHFR-LoxP-CaM1-2xTy-EF1/2m | *LoxP-DHFR-TS:DHFR-LoxP, CaM1:CaM1^D38A,D40A,D42A,D120A,D122A,D124A^-2Ty* | Complementation |
| p28 | pDHFR-LoxP-CaM2-2xTy | *LoxP-DHFR-TS:DHFR-LoxP, CaM1:CaM2-2Ty* | Complementation |
| p29 | pDHFR-LoxP-CaM2-2xTy EF1m | *LoxP-DHFR-TS:DHFR-LoxP, CaM1:CaM1^D13A,D15A,D17A^-2Ty* | Complementation |
| p30 | pDHFR-LoxP-CaM2-2xTy EF2m | *LoxP-DHFR-TS:DHFR-LoxP, CaM1:CaM1^D83A,D91A^-2Ty* | Complementation |

^1^ SAG1 promoter driving expression of Cas9-GFP.
