# Supplementary material for: Calmodulin-like proteins localized to the conoid regulate motility and cell invasion by Toxoplasma gondii
Source: PLoS Pathog. 2017 May 5;13(5):e1006379. doi: 10.1371/journal.ppat.1006379 (PMC5435356; doi:10.1371/journal.ppat.1006379)
Supplement: S3 Table — (DOCX) [file ppat.1006379.s004.docx]

**S3 Table**. Primers used.

| Primer name | Sequence (5'….3')^1^ |
| --- | --- |
| Set 1 | ATCGATAAGCTTATAACTTCGTATAGCATACATTATACGAAGTTATCAGCACGAAACCTTGA |
|  | TGGCGGCCGCATAACTTCGTATAATGTATGCTATACGAAGTTATGTGGATCCCCCTCCAC |
| Set 2 | GCCCGCTAGCAAGGGCTCGGGCTCGACCCAGCTGTAC |
|  | TCGACCCTCGAGTAGAACTAGTGGATCCGAGCAC |
| Set 3 | TAACCCGGGCATATGTAGAAAAG |
|  | GGCGCGCCcCGATTTAATTAAtccAGCGTAATCTGGAACG |
| Set 4 | TCGgGGCGCGCCGGCTCTGGCGGCGGCGGCGGCGGCTCTTACCCGTACGACGTC |
|  | TCGACCCTCGAGTAGAACTAGTGGATCCGAGCAC |
| Set 5 | GAGGTCCACACGAACCAGGACCCGCTCGATTAACCCGGGCATATGTAGAAAAG |
|  | ATCGAGCGGGTCCTGGTTCGTGTGGACCTCCAGCTGGGTCGAGCCC |
| Set 6 | GAACAAAAATTAATCTCAGAAGAAGACTTGTAACCCGGGCATATGTAGAAAAG |
|  | GCCCGAGCCCTTGCTAGCGAATTCCCGTCCTCCACTTC |
| Set 7 | GCCCGCTAGCAAGGGCTCGGGCTCGACCCAGCTGGACAAGGACAACACCG |
|  | CGACCCTCGAGTAGAACTAGTGGATCCGA |
| Set 8 | ATTCGGGCCCGCTAGCAAGGGCTCGGGCTCGACCCAGCTGATGGGCAGTGTCGAGC |
|  | AGAGCCGGCGCGCCcAGCTCTGCTCTTGCAC |
| Set 9 | TAACCCGGGCATATGTAGAAAAGTTG |
|  | ATCGAGCGGGTCCTGGTTCGTGTGGACCTCGCCGCCAGAGCCGGCGC |
| CAM1 sgRNA 3’ | CGCGAATAAATAAACTCGAGGTTTTAGAGCTAGAAATAGC |
| CaM2 sgRNA 3’ | CTGCAGAAAGTCTGACACCGGTTTTAGAGCTAGAAATAGC |
| CaM3 sgRNA 3’ | GTCGTTTGCAACGTTAGGTGGTTTTAGAGCTAGAAATAGC |
| MyoH sgRNA 3’ | CCGTGGTGTTGCGTGTCTGGGTTTTAGAGCTAGAAATAGC |
| SAS6L sgRNA 3’ | CTGGATGGAGCATAGATCAGGTTTTAGAGCTAGAAATAGC |
| CaM1 sgRNA 5’ | CCACCAGCGAGGACTCACGGGTTTTAGAGCTAGAAATAGC |
| CaM2 sgRNA 5’ | TGCGAGAACGCGGAAGGCAGGTTTTAGAGCTAGAAATAGC |
| CaM3 sgRNA 5’ | TCACGACGACGAGGAACACCGTTTTAGAGCTAGAAATAGC |
| sgRNA R | AACTTGACATCCCCATTTACCAG |
| Set 10 | CGAATTGGGTACCCAAGTAAGCAGAAGCACGCTG |
|  | TCGAC*CTCGAG*AATTAACCCTCACTAAAGG |
| CAM1 HR1-L | TTCTGTGGACTCGTGCAGGCGTCTCTGCCTTCCGCGAATAAAGCTAGCAAGGGCTCGGG |
| CAM1 HR2-T | CCTTGTGCAACCTTTCGCAGGCGCGTACGTTGTTTCCTCTCATACGACTCACTATAGG |
| CaM2 HR1-L | ATTCCCGACGTCGCCCAGAAAGTCGAGCAGCTGCAGAAAGTCGCTAGCAAGGGCTCG |
| CaM2 HR2-T | TGGCGACAGCGCGAGGCCTCCTTCTGGTTCCAGTCTCCACGGATACGACTCACTATAGG |
| CaM3 HR1-L | CGGTTTGCCGATTTCTGTGCCATATTCAGTCGTTTGCAACGTGCTAGCAAGGGCTCGG |
| CaM3 HR3-T | AATCGCTGGTCCACGTAACATGCTCAGACAGTAACCCCGCACAATACGACTCACTATAGG |
| MyoH HR1-L | CGTCCGTCGGTGAATCCGAACTGGGATCCCATGGCCTACAACGCTAGCAAGGGCTCGG |
| MyoH HR2-T | GGCACTCTGTTTCCCTTTTGGGATTCACAATGTAGGCCGCCAATACGACTCACTATAGG |
| SAS6L HR1-L | CGAGCTCCAGGAACCACGGAGGGCGCATCCACTCGGTTCCTCGCTAGCAAGGGCTCGGG |
| SAS6L HR2-T | GTCTCTTAGTGAGAAATAGAAAAGTCTGGATGGAGCATAGATATACGACTCACTATAGG |
| CaM1 M | CGTTTCCACCAGCGAGGACTCAGTAAAACGACGGCCAGT |
| CaM2 M | AAAATACGCGGAAGCCGAGTCTTGGTGCGAGAACGCGGAAGGGTAAAACGACGGCCAGT |
| CaM3 M | TCCCTGTCGCGTTCGTCTCACTGGCTCACGACGACGAGGAACCGACGTTGTAAAACGACGG |
| CaM1 p1 1p1 | GCCCAGAATGTTGAGCCTG |
| CaM1 p2 1p2 | GTGGGATGCGCTTCTGTG |
| CaM1 p3 1p3 | GATGGGAAGTTGAGTGAGGC |
| CaM2 p1 2p1 | CGATGGAAAGCAGACGAAC |
| CaM2 p2 2p2 | CGCATACACACAGACACG |
| CaM2 p3 2p3 | CGGTGTAACGCATGGGGAAC |
| CaM3 p1 3p1 | GTGAGTGCACCCGGTAGC |
| CaM3 p2 3p2 | CAACGGCGAACTGGAGGC |
| CaM3 p3 3p3 | CAGCTATCGCCAGCGGATG |
| p | CCCTCGAGTAGAACTAGTGG |
| Set 11 | GACGAGCTGTACAAGTAAGCT |
|  | ACCGACAGCCATCTCCATC |
| Set 12 | GGTTCGGCGGCCGCCCTAGGGCTAGCGGGCCCGAAGCTGCCCGTCTCTC |
|  | ACTATAGGACTAGTGGATCCCCCTCCACCGC |
| Set 13 | CCTATAGTGAGTCGTATTATGTGAAATTGTTATCCGCTC |
|  | ACTAGTCAGGAAACAGCTATGAC |
| Set 14 | GGTTCGGCGGCCGCCTGTGGTCGACGCAGAAGT |
|  | CCTTGCTAGCGGGCCCTTTATTCGCGGAAGGCAGAGAC |
| Set 15 | GCAGCGGCGGGAGCGGGGAAGTTGAGTGAGGCAG |
|  | GATTCTGGTGAAAATCGTTTCTAG |
| Set 16 | GCGACGGCGAGAGCGGGAAAATTGACCGCCGGC |
|  | GACGTACTGGAAAACTTCACG |
| Set 17 | GGTTCGGCGGCCGCCTGTGGTCGACGCAGAAGT |
|  | TTTGTCCCTAGGTGTGCAAATAAGGGAAAAACACG |
| Set 18 | TTGCACACCTAGGGACAAAATGGCGAACAATCTGGAG |
|  | GCTAGCGGGCCCGACTTTCTGCAGCTGCTCG |
| Set 19 | GAAGACTTTGAAGATGCTCTCCAG |
|  | GCGAGCGCGGGAGCgGGAAAGTTGACCTGCATCG |
| Set 20 | GCGCCGGCGAAGAAGGGTGTGGTGGATGcGCGGTCTCTGAACTATTTGTTG |
|  | GACGACGGAAAAGAGAGGG |

^1^ For primer sets, the top line is the forward primer and the bottom line is the reverse primer.
