# Supplementary figures and images for: Calmodulin-like proteins localized to the conoid regulate motility and cell invasion by Toxoplasma gondii
Source: PLoS Pathog. 2017 May 5;13(5):e1006379. doi: 10.1371/journal.ppat.1006379 (PMC5435356; doi:10.1371/journal.ppat.1006379)

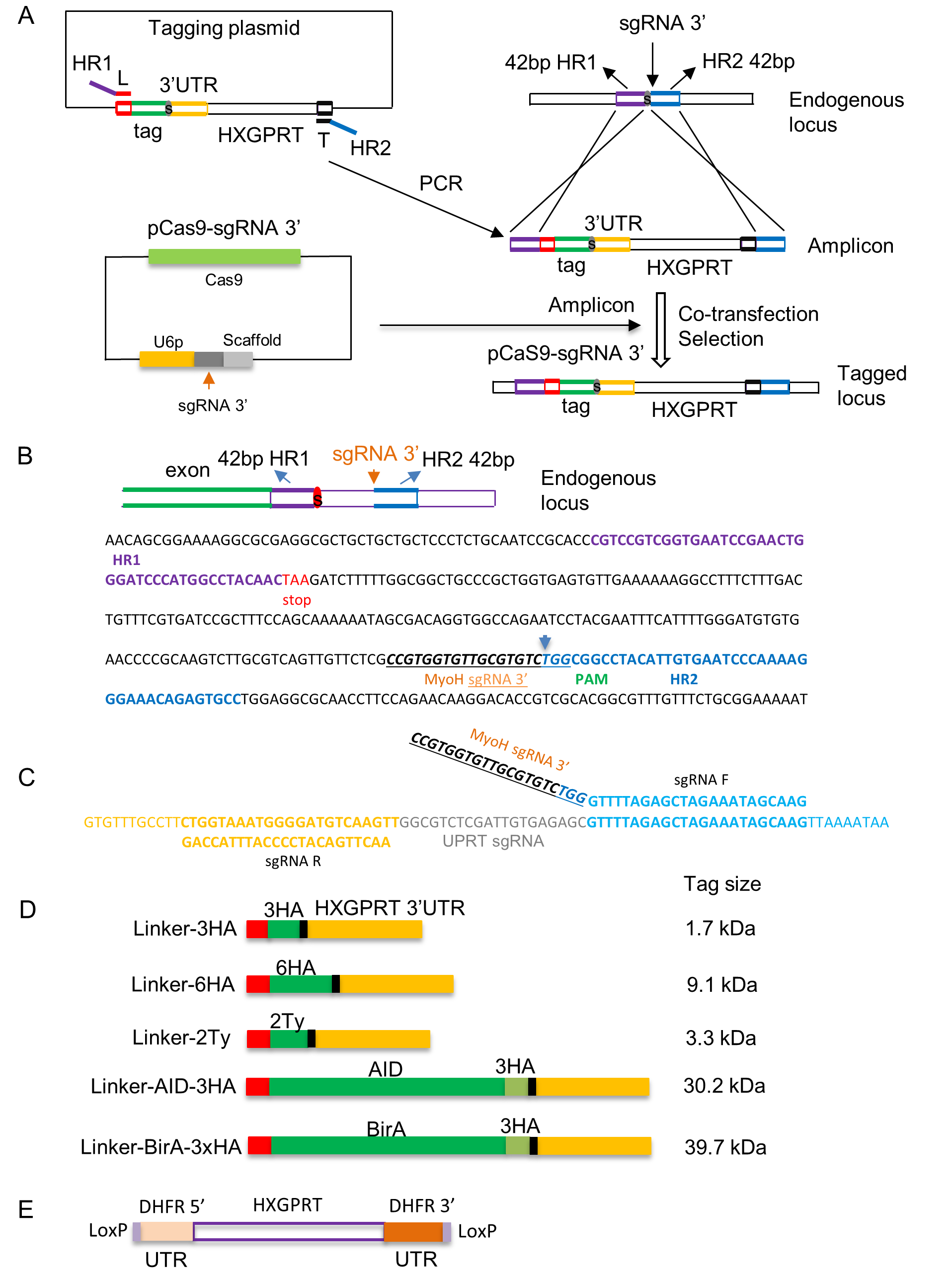

Supplement: S1 Fig — A. Schematic illustration of CRISPR tagging technology developed for T. gondii. Generic tagging plasmids that served as PCR templates for generation of gene-specific amplicons contained a linker (L, red box) and tags (green box) followed by a generic stop codon (gray box with s) and the HXGPRT 3’UTR (yellow box). A resistance marker expression cassette encoding HXGPRT, flanked with loxP sites, was included in the tagging plasmids, as illustrated. Amplicons were generated with a pair of primers incorporating the short homology HR1 (purple for the endogenous locus and red for the L region matching the Linker in the forward primer) and short homology HR2 (blue for the endogenous locus and black for the T region matching the T7 promoter (black) in the reverse primer) for a gene of interest. A Cas9-sgRNA 3’ plasmid that targeted close to the stop codon (gray box with s) of a specific gene of interest was combined with a gene-specific amplicon and co-transfected into a recipient line and transformants were selected with MPA and Xa as described in the methods. S in the gray boxes indicates a stop codon; U6p, RNA U6 promoter; scaffold, sgRNA scaffold; 3’UTR, HXGPRT 3’UTR. B. Example for the design of sgRNA 3’ and short homology region amplicons (HR1, HR2) for the gene encoding MyoH. The location in the sequence of the HR1 is shown in purple, the HR2 region in blue, and the sgRNA 3’ in orange. The Cas9 cleavage site is marked with a blue arrow. The stop codon is indicated by red lettering. C. Generation of a Cas9-sgRNA 3’ plasmid using Q5 DNA mutagenesis. A Cas9-sgRNA plasmid targeting the UPRT gene served as a DNA template for the Q5 mutagenesis reaction. The forward primer (sgRNA F) incorporated the MyoH sgRNA 3’ region and an adjacent region matching the sgRNA scaffold. The reverse primer (sgRNA R) was located just outside of the UPRT sgRNA on the reverse strand. D. Schematic of a variety of tagging plasmids used here. Different tags were integrated between the Linker and [file ppat.1006379.s006.tif]

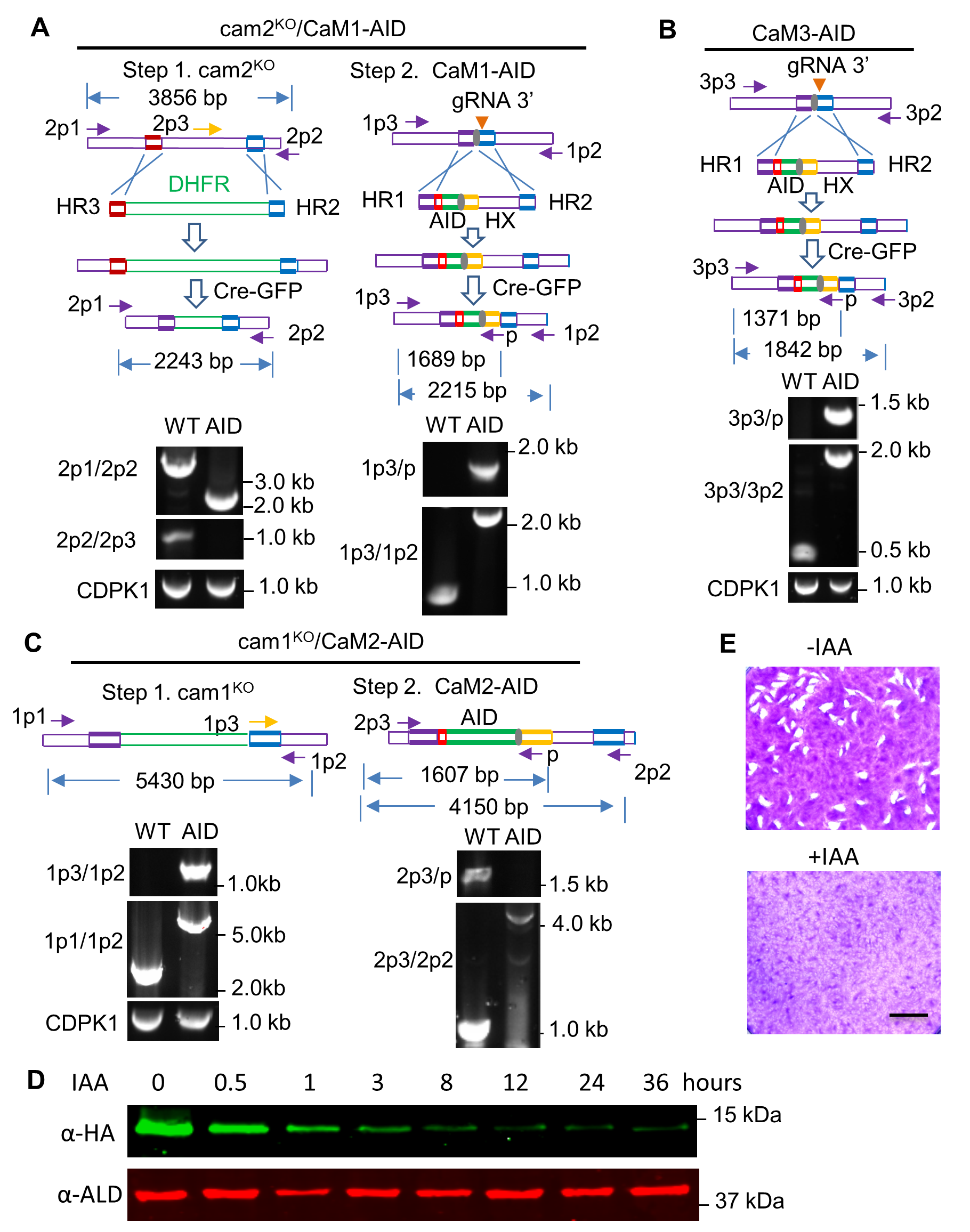

Supplement: S2 Fig — A and B. Schematic of generation and diagnostic PCR for validating clones of cam2KO/CaM1-AID (A) and CaM3-AID lines (B). The cam2KO/CaM1-AID line (A) was generated first (step 1) by deletion of CaM2 using the double sgRNA strategy in the TIR1 parental line followed by (step 2) CRISPR tagging at CaM1 C-terminus with AID. The CaM3-AID line (B) was generated by CRISPR tagging at the C-terminus with AID. The resistance markers encoding DHFR or HXGPRT were excised by transfection of a Cre-GFP plasmid. Diagnostic PCR was performed using primers shown in the diagram that includes the PCR product sizes. WT, the TIR1 parental line; AID, cam2KO/CaM1-AID (A) and CaM3-AID (B); CDPK1 was used as PCR control. C. Generation and verification of the cam1KO/CaM2-AID line using a similar strategy to that described above, except cam1 was deleted and CAM2 was tagged with AID. Diagnostic PCR was performed using primers shown in the diagram that includes the PCR product sizes. WT, TIR1 parental line; AID, cam1KO/CAM2-AID. D. Degradation efficiency of AID-tagged CaM2 in the cam1KO/CaM2-AID line cultured for different periods of time in media with addition of auxin (500 μM) or 0.1% ethanol (vehicle). Samples were resolved with SDS-PAGE, blotted and detected with anti-HA (mouse) and anti-aldolase (rabbit), and probed with Licor IR-dye conjugated secondary antibodies. E. Plaque formation by the cam1KO/CAM2-AID line grown in D10 culture medium containing auxin (500 μM) (+IAA) or 0.1% ethanol (-IAA). Parasites were grown for 7 days, stained with Crystal violet, and plates scanned to generate the image. Scale bar = 0.5 cm. (TIF) [file ppat.1006379.s007.tif]

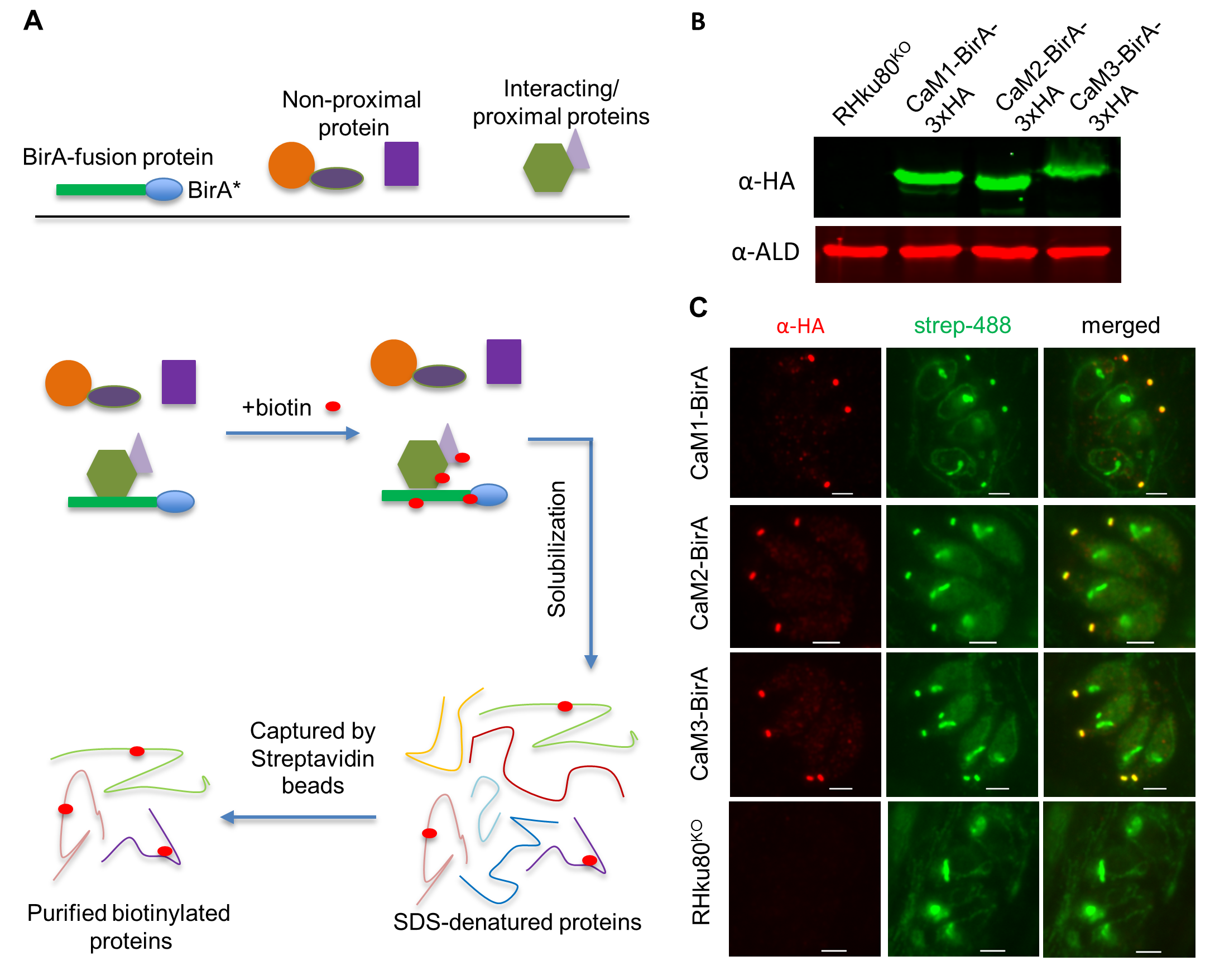

Supplement: S3 Fig — A. Fusion of BirA tag with endogenous protein labeling of interacting or proximal proteins in the presence of exogenous D-biotin in media. Biotinylated proteins were affinity purified using streptavidin conjugated magnetic beads from SDS-denatured lysis, and identified by mass spectrometry. B. Western blot confirmation of BirA fusion lines of CaM1-BirA-3xHA, CaM2-BirA-3xHA, and CaM3-BirA-3xHA. Western blot was detected with antibodies mouse-anti-HA (HA) and rabbit anti-aldolase (ALD), followed with Licor IR-dye conjugated secondary antibodies. C. Immunofluorescence confirmation of biotinylation in CaM1-BirA, CaM2-BirA and CaM3-BirA lines. Parasites were grown in media containing D-Biotin for 24 hr, fixed, permeabilized, and stained with antibodies rabbit anti-HA, followed with anti-rabbit Alexa Fluor-594 and streptavidin-Alexa Fluor-488. The parental line ku80KO served as a control. Endogenous biotin containing proteins were detected in all lines, while apical CaM-dependent labeling was only seen in the BirA fusion lines. Scale bar = 2 μm. (TIF) [file ppat.1006379.s008.tif]

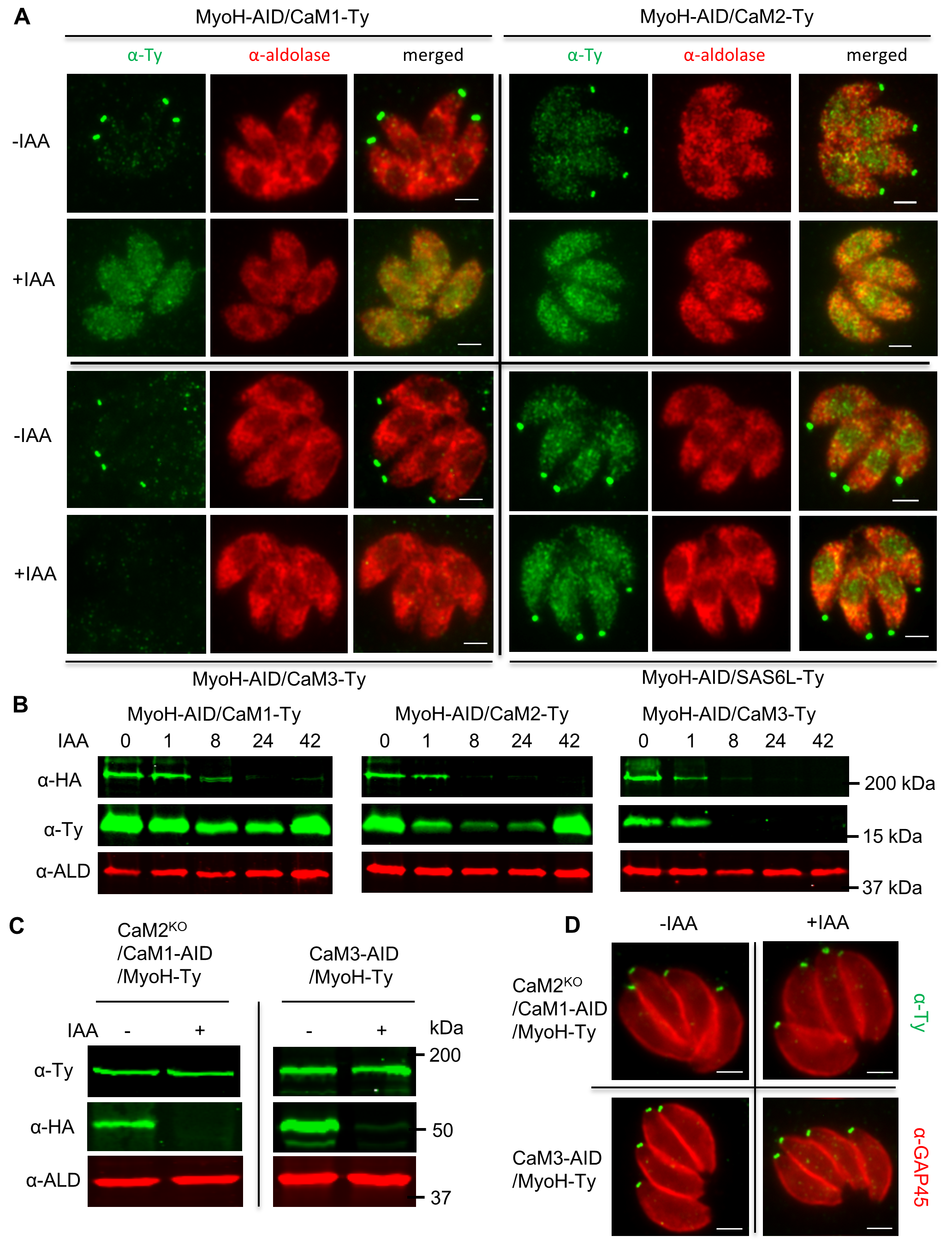

Supplement: S4 Fig — There was a loss of conoid localization or disappearance of CaMs upon depletion of MyoH (A, B). In contrast, MyoH was stable in the CaM2KOCaM1-AID and CaM3-AID strains following auxin degradation (C, D). A. CaM1, CaM2 and CaM3 were tagged with 2xTy in the MyoH-AID line. Parasites were grown on coverslips with HFF for 8 hr with 500 μM IAA (+IAA) or ethanol alone (-IAA), fixed, and stained by IFA using mouse anti-Ty antibodies and rabbit anti-aldolase antibodies followed by anti-mouse Alexa Fluor-488 and anti-rabbit Alexa Fluor-594. The protein SAS6L was used as control. Scale bar = 2μm. B. Western blot detection of CaMs tagged with Ty in the MyoH-AID line. Parasites were grown for different treatment times with IAA (500 μM) or 0.1% ethanol (vehicle). Parasites were resolved with SDS-PAGE, blotted, and probed with mouse anti-HA, mouse anti-Ty, and rabbit anti aldolase, followed by Licor IR-dye conjugated secondary antibodies. C. MyoH was tagged with 2Ty in the AID strains. Parasites were treated with either IAA (500 μM) or 0.1% ethanol (vehicle) for 2 days and Western blotted with anti-HA (to detect the AID fusion), anti-Ty (to detect MyoH), and anti-aldolase (ALD, control) antibodies followed by Licor IR-dye conjugated secondary antibodies. D. Immunofluorescence microscopy was performed with parasites grown for 24 hr with either IAA (500 μM) or 0.1% ethanol (vehicle) and stained using anti-Ty (stained green) and anti-GAP45 (stained red) antibodies. Scale bar = 2μm. (TIF) [file ppat.1006379.s009.tif]
